# Fluid injection interruption causes temporary changes in local stress field and induced seismicity at Krafla caldera, Iceland

**DOI:** 10.1038/s41598-026-39532-1

**Published:** 2026-02-26

**Authors:** Elisabeth Glück, Roberto Davoli, Thorbjörg Ágústsdóttir, Stéphane Garambois, Egill Árni Gudnason, Yan Lavallée, Anette K. Mortensen, Bettina Scheu, Jean Vandemeulebrouck

**Affiliations:** 1 Univ. Grenoble Alpes, Univ. Savoie Mont Blanc, CNRS, IRD, Univ. Gustave Eiffel, ISTerre, Grenoble, France; 2 Ludwig-Maximilians Universität München, Munich, Germany; 3 Iceland GeoSurvey (ÍSOR), Kópavogur, Iceland; 4 Landsvirkjun, Reykjavík, Iceland

**Keywords:** Induced seismicity, Seismic anisotropy, Shear-wave splitting, Fault reactivation, Geothermal energy, Krafla volcano, Natural hazards, Solid Earth sciences

## Abstract

Induced seismicity related to fluid injection in the upper crust is a major concern in the context of geothermal energy production. For exploited high-temperature geothermal systems in tectonically and volcanically active areas, such as Krafla caldera in NE Iceland, understanding the processes that trigger seismicity and changes in the local stress field can be difficult to unravel. We observe a link between anthropogenic activity and changes in the local stress field, since the appearance of a strike-slip cluster coincides with changes in the seismic anisotropy around an injection well during an injection interruption period. By analyzing the shear-wave splitting phenomenon, 90$^\circ$ flips of the fast S-wave polarization and a decrease in the time delays between the fast and the slow S-wave component is observed, starting within hours after an injection stop, coinciding with a sharp increase of strike-slip events in the vicinity of the well. When the injection restarts, the seismic quiescence and increase of time delays may suggest a resumption of the previous state. The changes in seismicity patterns and variations of the seismic anisotropy might be linked to changes in the pore pressure and a possible activation of a shear-fault due to anthropogenic activity.

## Introduction

### Induced seismicity and seismic anisotropy variations related to stress changes

Induced seismicity associated with geothermal exploration during drilling, fluid injection and stimulation, especially in enhanced geothermal systems^1^, is a major societal concern that hinders its exploitation despite the need for carbon-free energy. Although most geothermal induced seismicity is of small magnitude, several larger events have been felt and, in some cases, have caused damage and financial loss (Basel (Switzerland) - $M_L$ 3.4^2^, Vendenheim (France) - $M_L$ 3.9^3^, Soultz-sous-Forêts (France) - *M* 2.9^4^, Hellisheidi (Iceland) - *M* 4^5^, Pohang (South Korea) - *M* 5.5^6^).

Fluid injection in geothermal reservoirs is commonly used to prevent large drops in pressure due to extraction, whilst renewing the resource. However, such injections induce temperature and pressure fluctuations, which modify the stress field and may induce rock-fluid interaction as well as seismogenic rupture^7^, depending on the injected rate and volume^8^. In this context, rupture can be generated by stress transfer due to changes in pore pressure and/or external (normal or shear) stress acting on a fault system^9,10^, notwithstanding the tectonic setting and local stress field^8,11^. In volcanically and/or tectonically active areas, the origin of earthquakes – anthropogenic vs. natural – is difficult to discriminate and correlation becomes more credible when seismicity is occurring closely in time and space to the injection (e.g.^8,12,13^). In seismically quieter areas, it has been observed that induced seismic events can be triggered during injection as well as, arguably, years later^14^. For large injected volumes, the changes in stress associated with injection may extend over long distances, generating distal seismic events up to tens of kilometers away from the injection well^11,15,16^. Following fluid injection and/or thermo-mechanical stimulation, the interruption of fluid injection induces further changes in the local stress field that may also prompt induced seismicity^17–20^, yet such occurrences are less commonly documented. Ultimately, a robust understanding of the cause of seismicity in volcanically and/or tectonically active areas exploited for geothermal energy, is required to resolve the impact of anthropogenic manipulation and alleviate the risk. This comes as a necessary requirement for the safe exploitation of shallow crustal resources (geothermal, ore, etc.) and the safe development of future in-situ magma observatories^21^.

Seismicity is a direct result of the dominant stress field. The primary metric to constrain stress, apart from borehole breakouts at a very local scale, is the analysis of earthquake focal mechanisms^22^. Another powerful tool to retrieve the main stress direction is the shear-wave splitting (SWS) phenomenon associated with the passage of a wave in an anisotropic system^23–25^.[When an S-wave propagates through such a medium, it splits into two components, perpendicular to each other, with the polarization direction of the fast component ($\varphi$) being parallel to the maximum horizontal stress ($S_{Hmax}$). The travel time delay between the two components ($t_d$) is an indicator of the intensity of the seismic anisotropy^23,25,26^.] Crustal seismic anisotropy may be imparted by the reservoirs lithostratigraphic structure (e.g. layering, rock types, and orientation) as well as by stress^23,27^. When anisotropy is stress induced, the fractures align parallel to $S_{Hmax}$, thus promoting anisotropy of the permeable porous network^24,28^ and redistributing fluids^29^. The presence of fluids in the aligned micro-cracks leads to increased $t_d$^18,30,31^, and if the pores are saturated, $\varphi$ flips by 90$^\circ$, as pore pressure pushes against fracture surfaces perpendicular to $S_{Hmax}$^32,33^. Thus, SWS can be used as an indicator for fluid saturation and pore pressure changes. However, SWS analysis is prone to wide scattering in assessing $\varphi$ and $t_d$, since accurately identifying and measuring the first onset of S-waves can be challenging. This is the main reason why SWS is generally considered less reliable in resolving the stress than the focal mechanism approach, which is based on the polarity of the P-wave^22^. However, it offers an important constraint on the anisotropy of the system, which reflects the presence of fluids, a central issue when studying geothermal systems.

Active volcanoes exploited for geothermal energy production are exposed to significant stress fluctuations due to tectonic, magmatic, hydrothermal and anthropogenic processes. Such variations may occur over different time scales, ranging from seconds to centuries. Inflation and deflation processes due to magma transport can influence the stress field close to seismogenic regions (imprints in focal mechanisms)^34,35^, as well as in the surrounding medium, where variations in the stress field induce seismic anisotropy changes^36–38^. While internal processes (e.g. magma, pressurization, etc.) generally affect larger and deeper parts of the volcano, external processes (e.g. glacial loading^39,40^, geothermal operations or hydrothermal activities) tend to locally alter the stress field close to the surface. For instance, fluid injections in geothermal systems have resulted in: i) decrease (e.g. Krafla, Iceland) as well as increase (e.g. Coso, US) in seismicity^17,18^, ii) changes of the P-axis in focal mechanisms (e.g. Geysers, US^41^ or Krafla, Iceland^42^), and iii) changes in anisotropy and consequently $t_d$ values^17,43,44^. Over longer timescales and in rare cases, the 90$^\circ$ flip of $\varphi$ has been observed^31^. In volcanic settings, 90$^\circ$ flip of $\varphi$ is generally interpreted to reflect large-scale magmatic processes, in particular the movements of pressurized magma^33,45–48^. However, magma emplacement can locally heat and pressurize the surrounding hydrothermal systems, thus that the scale and extent of seismic anisotropy variations should be determined to interpret the nature of subsurface processes. With this in mind, we propose here SWS analyses on different spatial and temporal scales. By focusing on a short-term semi-controlled experiment of interrupted fluid injection at the central part of the Krafla geothermal system (NE Iceland) we assess the impact of such practice on the state of the local stress field.

### Natural and anthropogenic geothermal processes at Krafla caldera, NE Iceland

**Figure 1:**
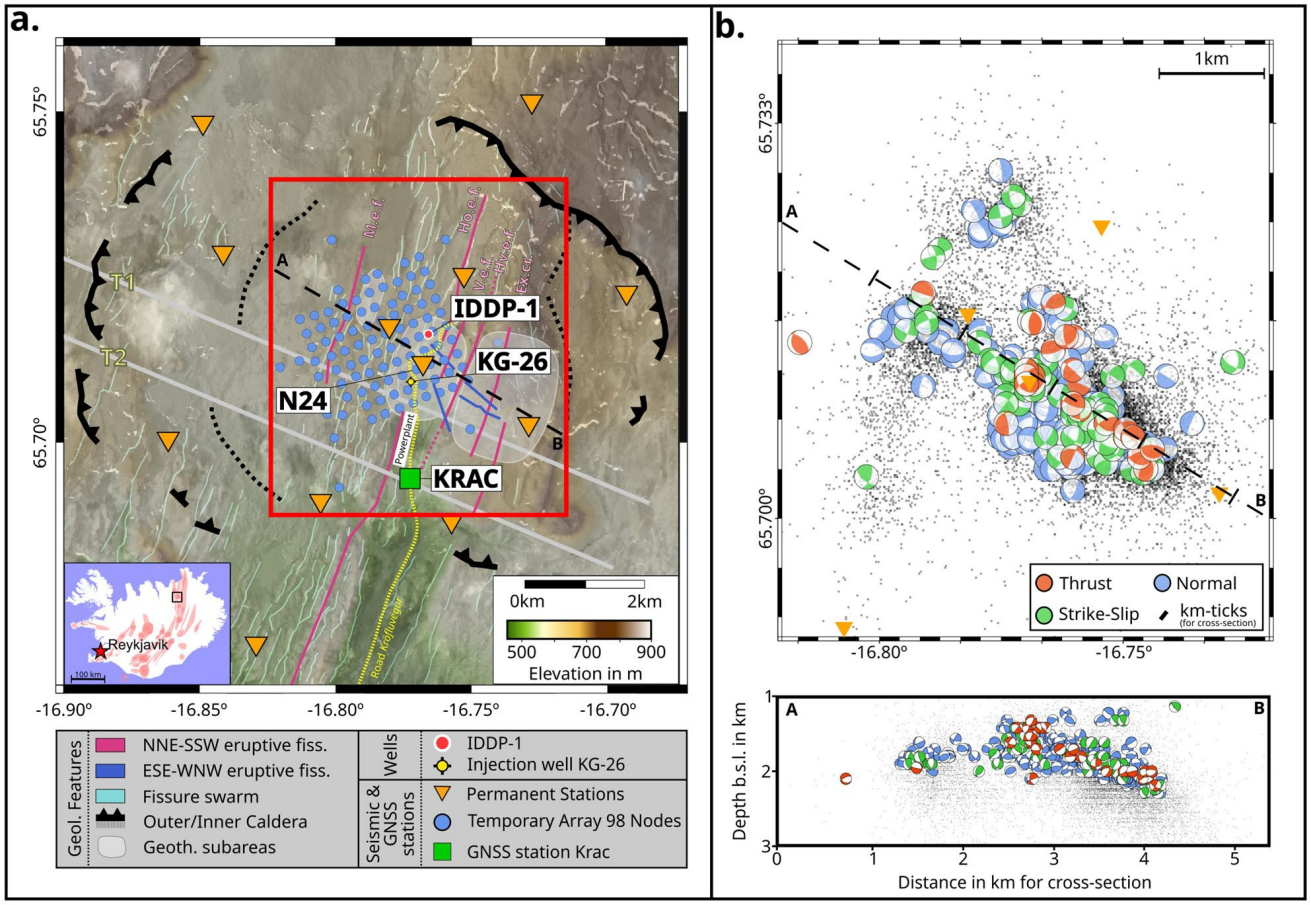
Overview of the Krafla caldera, the seismic deployments (maps were generated with PyGMT^67^), the seismicity and the available focal mechanisms: a) Black (solid, toothed) line shows the caldera rim, while pink and blue lines are eruptive fissures^55^. The thin turquoise lines mark the fissure swarm. The inner caldera (black dashed line) and the transverse structure (delimited by white lines, T1 and T2)^50^ are still debated. The large-scale (orange triangles) and small-scale (blue dots) seismic networks at Krafla (indicated node: N24) and the GNSS station KRAC (green square), with the location of the wells IDDP-1 and KG-26. Inset: Location of Krafla in Iceland^68–70^. Red box shows the location of b) with the black line indicating the location of the cross section. b) Top: Spatial distribution of the seismicity in the ÍSOR catalog during 2017 - 2022 (small black points), double-couple source mechanisms from 2018 to 2022^42^ (order of plotting: normal faulting (blue), strike-slip (green), thrust faulting (red)) and permanent seismic stations (orange triangles). Bottom: Cross-section of the distribution of seismicity and mechanisms with depth.

Krafla is a central volcano in northeast Iceland, 20 km in diameter, transected by a 90 km-long NNE-SSW trending fissure swarm (Figure 1a, thin turquoise lines) that results from the ESE-WNW extension that characterizes northern Iceland. The volcano has been active for the last 300.000 years, and an 8 - 10 km wide caldera developed approximately 110.000 years ago^49^. The presence of an inner caldera of around 80.000 years is still debated^50^. Recent volcanic activities have been associated with rifting events: the Mývatn fires occurred from 1724 to 1729^49,51^ and the Krafla fires from 1975 to 1984^52^. In the early 1970s, just before the most recent eruptive period began, the Krafla geothermal powerplant was constructed and thus the caldera has been closely monitored for both volcanological and industrial purposes over the last decades.

At Krafla, geothermal exploration has provided an almost unparalleled insight into the subsurface structure of the geothermal system and its fluids that reach up to 450$^\circ C$ at 2 km depth^53–55^. Storage capacity and fluid pathways are dictated by the lithology. The uppermost kilometer is mainly formed of extrusive volcanic rocks with some intrusions, whereas intrusive rocks dominate below 1 km^56,57^. The pore space has been progressively filled due to secondary mineral precipitation^55,58,59^ and is affected by compaction processes^60^. In 2009, during drilling of the first well from the Icelandic Deep Drilling Project (IDDP-1) to reach supposed supercritical fluids at 4.5 km depth (Figure 1a)^61,62^, drilling unexpectedly encountered a magma body at 2.1 km depth^63^, providing information about the heat source of this energetic resource. The power station currently generates 60 MW of electricity through up to 20 wells^55^; 1-2 wells are used as injection wells.

Krafla is tectonically, magmatically and hydrothermally active, contributing to local and/or regional seismicity. Since 2013, Iceland GeoSurvey (ÍSOR), operates a 12 station permanent seismic network at Krafla [hereafter referred to as large-scale network] for the Icelandic national power company, Landsvirkjun. For the period 2006 - 2022, ÍSOR has compiled an extensive seismic catalog of Krafla containing over 50.000 events^42^. A subset of this catalog for the period 2017 - 2022 was relocated with a recent 3D velocity model^64^, comprising about 22.000 earthquakes [This event catalog is hereafter referred to as the ÍSOR catalog]. Studies of focal mechanisms of earthquakes and SWS at the regional scale have provided insight into the stress field at Krafla^18,42,65,66^. A previous study^42^ analyzed over 640 focal mechanisms covering the time interval between 2018 and 2022 [referred to as FM catalog], showing a predominantly extensional regime with normal faulting events, oriented in different directions, interspersed with strike-slip and also thrusting events (Figure 1b). The dataset indicates stress rotation between 2020 - 2022 as activity shifted from N-S striking faults to NW-SE striking faults. Non-double-couple events have also been detected in the geothermal system and attributed to the presence of fluids^42,65,66^. A SWS study in 2004^18^ revealed a similar spatial distribution of orientations of $S_{Hmax}$, which is consistent with later findings^42^. During the 2004 seismic experiment^17,18^, fluid injection in well KG-26 (Figure 1a) was shut-off for 10 days, resulting in elevated seismicity as well as a decrease in $t_d$, but no changes were observed in $\varphi$.

We revisit this link between the stress field changes and fluid injection patterns here, using a multi-scale approach and analyze the temporal and spatial variability of the seismic anisotropy at Krafla, by integrating SWS, 3D Vp/Vs tomographic reconstruction and deformation data. This is possible thanks to high-resolution datasets: we used data from the large-scale network at Krafla (Figure 1, orange triangles) between 2017 and 2022, which, in June-July 2022, were augmented by a 25 day nodal seismic experiment counting 98 nodes, deployed at 250 m intervals (Figure 1a, blue circles). During this short period, the injection was shut off in well KG-26 for 3 days (between June 28 and July 1) in a single step over a time period of 30 minutes, following a wellhead pressure of 1.1 bar at an injection rate of 65 l/s. With the dense temporary seismic network, we could assess whether injection fluctuations would overprint natural stress variations, which should leave characteristic geophysical signatures, including the identification of previously undetected increases in seismicity or localized variations in seismic anisotropy. The multi-scale approach used here enables us to assess stability and changes in the regional stress field over a period of six years. The ”injection experiment” provides an opportunity to monitor and constrain the effects of fluid injection on the stress field, including magnitude and extent.

## Results

### Template matching and identification of shear fault seismicity

**Figure 2:**
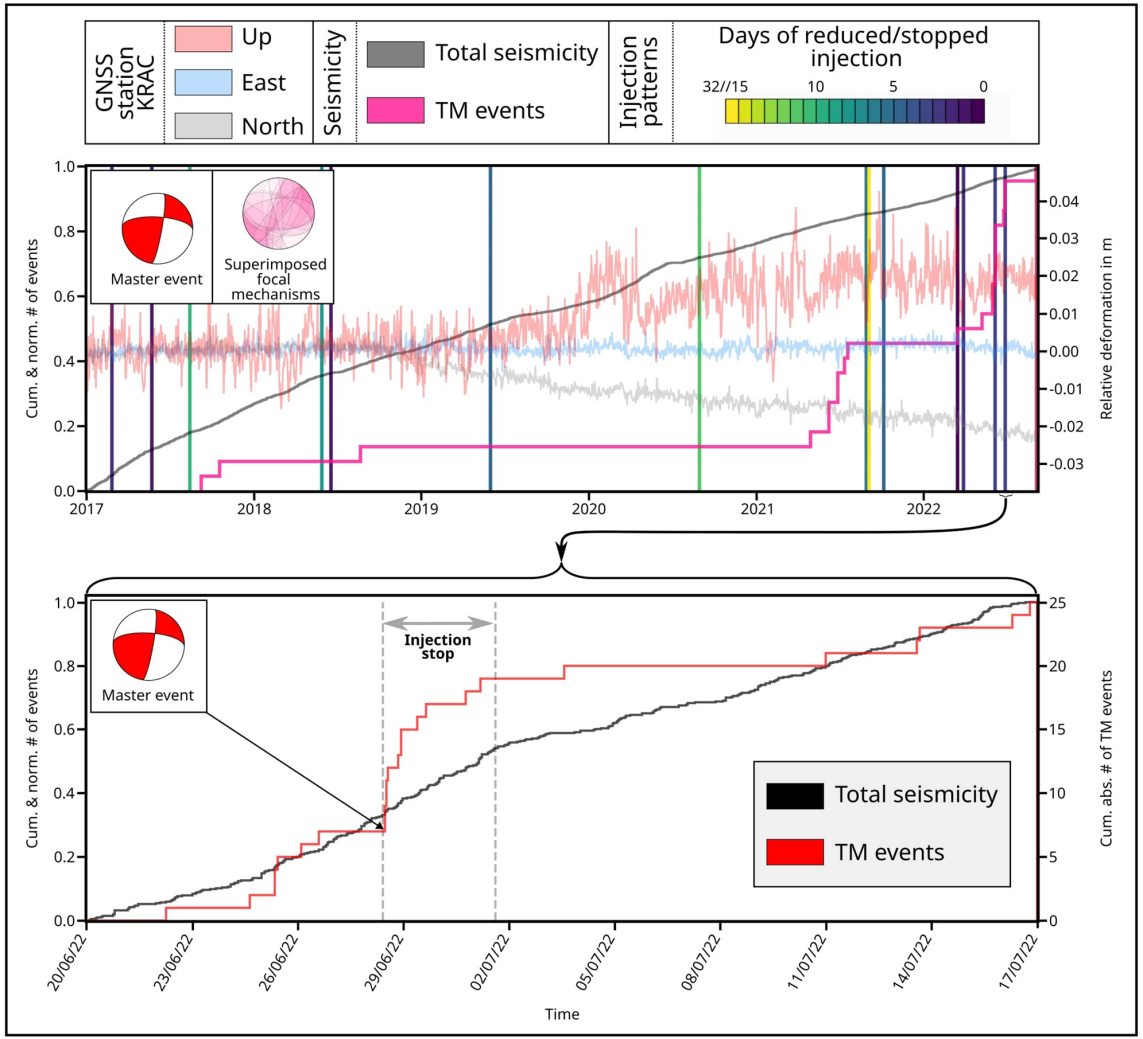
Long- and short-term evolution of seismicity patterns of the strike-slip events close to well KG-26. Top: Long-term evolution of the total seismicity (21.771 events, grey line), the strike-slip events found by template matching (TM) in the ÍSOR catalog (summed cc> 1.95 at the stations SPB, LHN and HVET5, 126 events, pink line), by using the master event, the three components of the GNSS station KRAC^71^ and the times of injection pauses at well KG-26. Inset with beachball plots: Red focal mechanism - Master event, pink superimposed mechanisms - TM results included in the FM catalog. Bottom: Short-term evolution of the total seismicity during the experiment in June and July 2022, with the detections of the temporary array (404 events, black line), the matching signals found by TM (cc > 0.5 at node N24, 25 events, red line) with the master event (arrow pointing to the occurrence time) on July 28 when the injection was paused (vertical dashed lines). The temporal evolution of the total seismicity is presented to highlight the differences compared with the trends observed in strike-slip events.

The deployment of the temporary nodal experiment allowed a higher detection rate of seismicity at Krafla^64^. These additional events are primarily located in the center of the caldera, with the same spatio-temporal evolution as the ones listed in the long-term ÍSOR catalog (Figure 2, top - grey line (total number of events: 21.771), bottom - black line (total number of events: 404)). A change in seismic patterns could be observed with the interruption of fluid injection. Within the first 24 hours of the injection pause, an anomalous increase in seismicity was observed near the injection well KG-26, compared to other areas (see Supplementary Figure s1). The first event appearing in this area (ID given by ÍSOR 2022mprrpt - June 28, 12:16:50 - $M_L$ 0.0, hereafter called “master event” and used as the reference event in the subsequent analysis) was detected below node N24 (Figure 1a). It is also listed in the FM catalog, showing a strike-slip source mechanism (Figure 2, red beachball plot). Cross-correlation of the waveforms of other nearby events with this master event exhibited correlation coefficients (cc) greater than 0.5 - which is a high value compared to what typically is observed at Krafla, due to the high degree of scattering. Using template matching at the permanent stations SPB, LHN and HVET5, 126 events with similar waveforms could be identified in the ÍSOR catalog from 2017 to 2022 (Figure 2, pink line). Ten of these events are listed in the FM catalog and also exhibit strike-slip behavior (Figure 2, superimposed focal mechanism inset). The temporal evolution of these particular swarm-like strike-slip events differs greatly from the overall, more diffuse, seismicity at Krafla. To determine the triggering mechanism of the strike-slip cluster, we compared its temporal evolution with corrected GNSS data from the station KRAC (continuous, daily measurements since 2012)^71^ and the periods of injection stops at the well KG-26. However, at first, no clear correlation between the appearance of the strike-slip events and deformation could be identified.

To improve our ability to resolve the spatio-temporal evolution of seismicity, we turn to a densified event catalog compiled for the time of the nodal experiment^64^ (see Supplementary Figure s2 for location). This enhanced dataset shows a temporal correlation between the injection interruption and the occurrence of the strike-slip cluster. To identify signals of seismic events too weak to be located with the dense nodal array, one node, N24, located directly above the strike-slip cluster, was used for template matching (for the waveforms of the master event at N24 and the newly identified events see Supplementary Figure S3). Using this method, 25 signals with cc > 0.5 were detected during the 25-day course of the experiment; 11 of which occurred within the first 80 hours after injection stopped. It is notable that post-injection seismicity began abruptly and then gradually ceased, with almost no events detected after three days, even once the injection resumed (Figure 2, bottom - red line). Based on this observation in July 2022, we opted to perform template matching using the same master event at closest station from the permanent network (HVET5). By using a lower correlation coefficient threshold of 0.5 (which may likely include more false detections), we observe a temporal correlation between strike-slip seismicity and every injection pause undertaken between 2017 and 2022 (Supplementary Figure S4). Thus, we proceed to perform a stress analysis, using SWS, to identify the underlying conditions and the cause of this cluster.

### Large-scale SWS analysis using the permanent network

**Figure 3:**
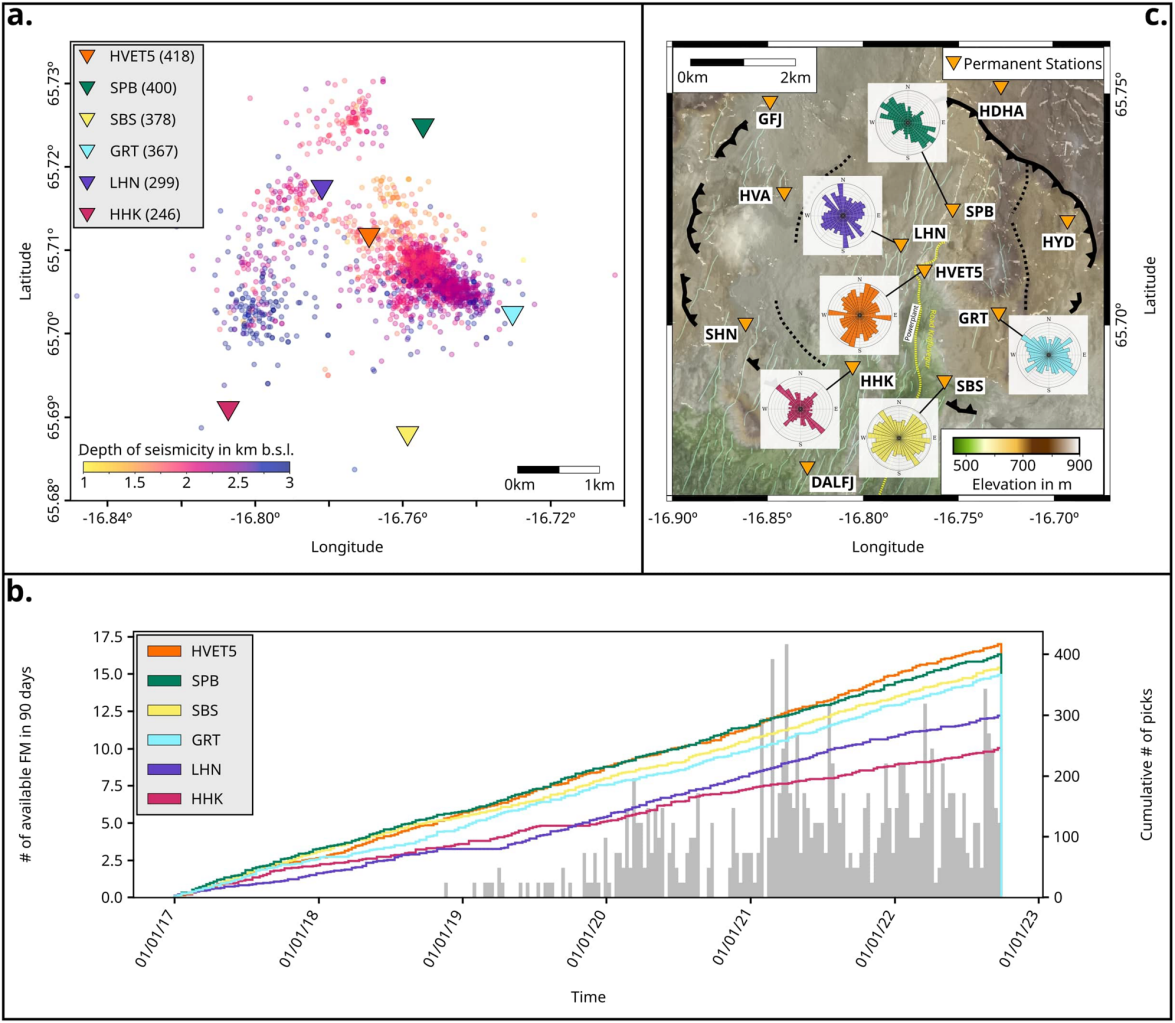
Database for the large-scale SWS. a) Spatial distribution of events used for the SWS (colored with depth), number of picks per station in brackets used to analyze the seismic anisotropy. b) Cumulative histograms - temporal distribution of picks for each station, grey histogram - temporal distribution of DC events provided in the FM catalog. c) The rose diagrams show the orientation of $\varphi$ derived from SWS at the 6 permanent stations (orange triangles) that were analyzed. The binning in the diagram is 10$^\circ$ and each circular grid line equals 1 pick. The map was generated with PyGMT^67^.

Initially, we performed SWS analysis using the ÍSOR catalog, based on the large-scale network, which contains seismic events that originate mostly around 2-km depth (Figure 3a, b). Only waveforms with good signal-to-noise ratio (SNR) and clear S-wave onsets on the horizontal components, were used to perform SWS analysis and compute the polarization angle $\varphi$ and the time delay $t_d$. The selection of events used for each station was constrained by the incidence angle, which was limited to $<45^\circ$, so that the polarization angle is a representation of the anisotropy underneath the station. Thus, the SWS analysis did not always rely on the same set of events. By focusing only on the 6 permanent stations located in the center of the caldera, we obtained in total 2108 picks; the number of picks for each station is shown in Figure 3a,b.

**Figure 4:**
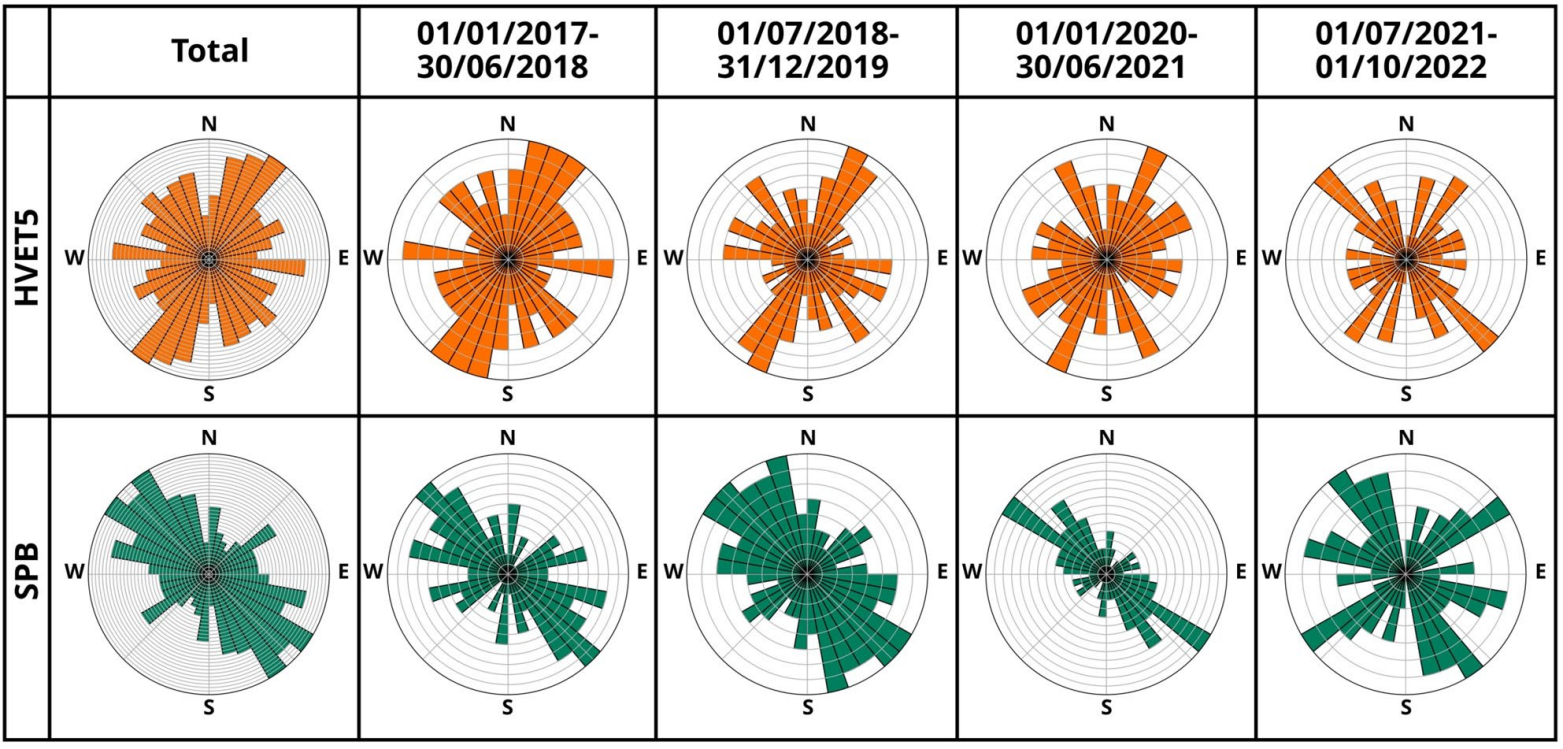
Time-lapse of the general stress field at two of the six analyzed permanent stations HVET5 and SPB, in 1.5 year steps (others are shown in Supplementary Figure S5). The binning in the diagram is 10$^\circ$ and each circular grid line equals 1 pick.

SWS analysis of permanent stations shows a dominant direction of $\varphi$ that changes locally, although scattering is often present. For the central part of the geothermal system, results agree with previous observation^18^ (Figure 3c). At LHN, the primary seismic anisotropy is almost due N-S, which corresponds to the orientation of the local fissure swarm. However, we also notice two additional seismic anisotropy directions, NNW-SSE and NE-SW. At station HVET5, we observed two dominant anisotropy directions: the first one aligns NNE-SSW with the rifting direction (as observed at LHN), while the other is oriented almost E-W. The GRT, SBS and HHK stations located in the southern part of Krafla all show a dominant NW-SE anisotropy direction, with contrasting secondary anisotropy in perpendicular directions. The SPB station in the north-east is the most stable, with the orientation of seismic anisotropy being oriented NW-SE, sub-parallel to the outer caldera boundaries, and no clear secondary direction. Since changes in the stress field have been observed in the strike of the focal mechanisms with time^42^, we analyzed the temporal evolution of the SWS direction. We used time steps of 1.5 years, which was the best compromise between number of events for the picked stations and time resolution (Figure 4, Supplementary Figure S5). During the 6 years of seismicity analyzed, we identified that most of the stations exhibit temporal changes in anisotropy direction, while 2 stations (SPB, HHK) remained constant over time (Figure 4). Due to the overlap of anthropogenic and natural processes on large time scales^36,72^, finding a clear correlation between changes in the stress field, induced seismicity and their causes requires a more detailed analysis. This was made possible by the deployment of the small-scale, high-resolution nodal network, during which time the injection was interrupted as an experiment between June 28 and July 1 2022, offering the opportunity to isolate the role and effects of fluid injection from natural processes.

### High-resolution SWS analysis during injection experiment

**Figure 5:**
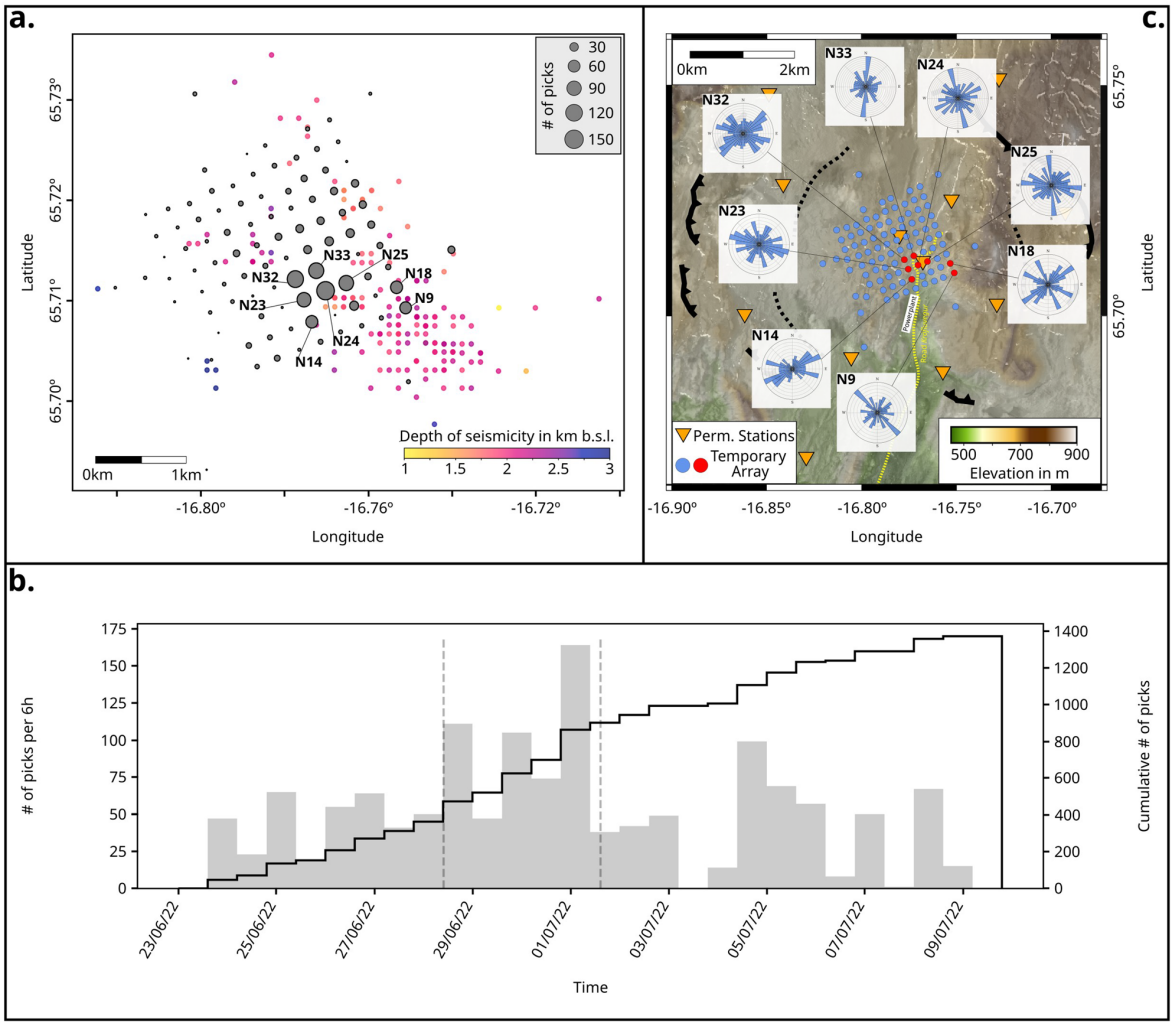
Database for the small-scale SWS. a) Spatial distribution of events used for the SWS (colored by depth), and location of the stations with sufficient picks (gray circles: node location, circle size representing the amount of picks at the respective station) to analyze the seismic anisotropy. b) Temporal distribution of picks at all stations. c) Rose diagrams (blue) showing the orientation of $S_{Hmax}$ derived from SWS at the 8 nodes (red points) analyzed. The binning in the diagram is 10$^\circ$ and each circular grid line represents 1 pick. Permanent seismic network (orange triangles), other temporary nodes (blue). The map was generated with PyGMT^67^..

**Figure 6:**
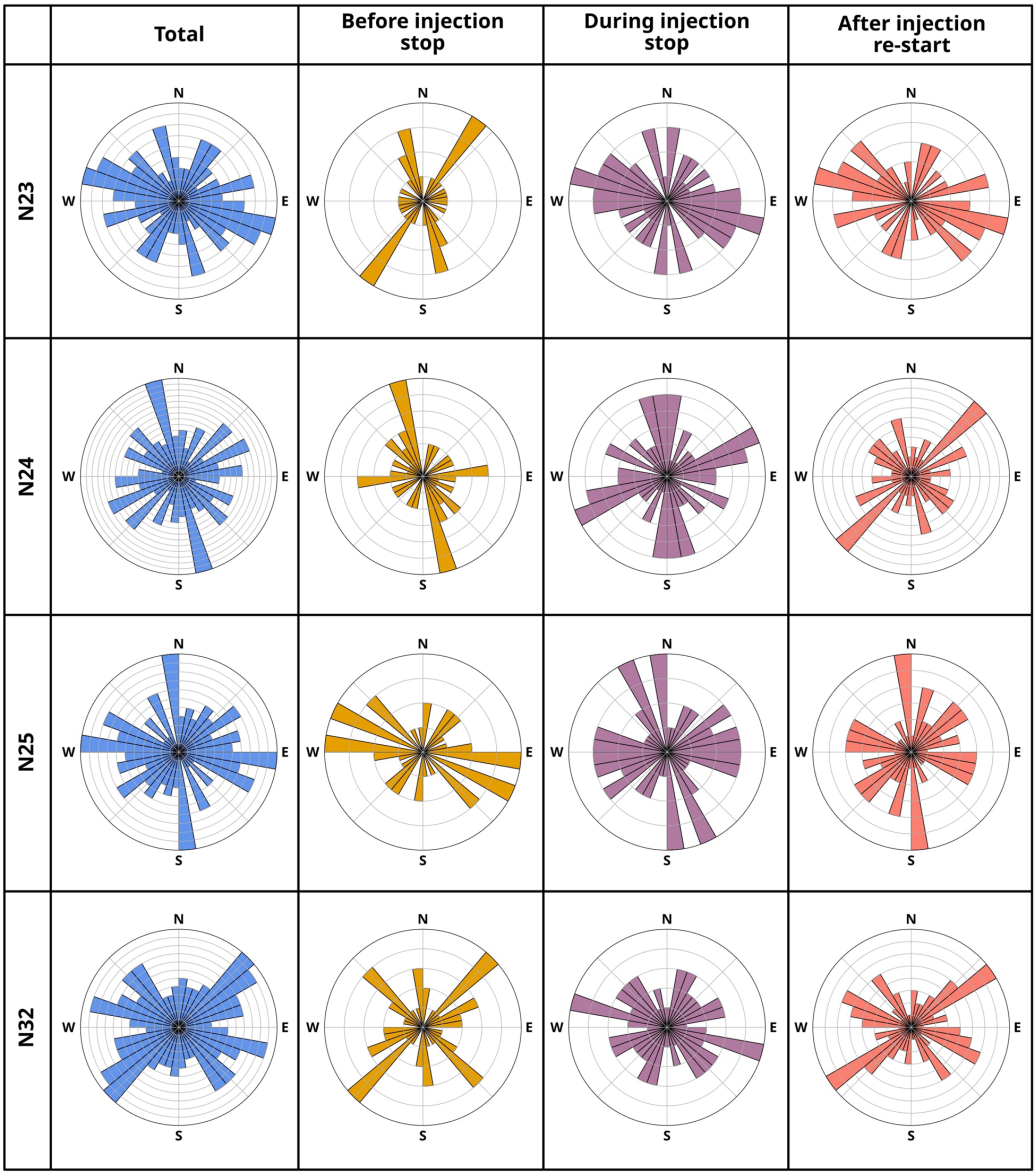
Time-lapse of the short-term experiment: Selection of 4 nodes N23, N24, N25 and N32 with the rose diagrams showing the orientation of $\varphi$. The binning in the diagram is 10$^\circ$ and each circular grid line equals 1 pick. Blue - Total dataset of $\varphi$, yellow - before the injection pause, purple - during the injection pause, red - after the injection re-start.

SWS analysis using 98, closely located seismic nodes, allows a clearer view of stress changes with fluid injection. Firstly, when considering the entire dataset (in total 1497 picks, with the number of event-station pairs per station shown in Figure 5a, b), it can be seen that the orientation of $\varphi$ between each node is often variable (see Supplementary Figure S6), even at a mere 250 m spacing. Node N33 shows a very clear N-S orientation of polarization of the fast component, similar to the nearby permanent stations LHN and HVET5 (Figures 3c, 5c). The N24 and N32 nodes show dominant polarization directions of stress perpendicular to each other; at N24, $\varphi$ is oriented NNW-SSE and ENE-WSW and at N32 it is NW- SE and NE-SW. Nodes N23, N25 and N14 have less clearly defined polarization directions, with three or more dominant angles. Nodes N18 and N9 located to the west in a different subsystem of the Krafla geothermal field, also show multiple dominant directions of $\varphi$, as observed at the nearest stations, HVET5 or GRT. However, these two stations also exhibit polarity in the NNW-SSE direction. Secondly, we explored how $\varphi$ and $t_d$ vary before, during, and after the 3-day interruption of the injection at the KG-26 well (Figure 5b, vertical dashed lines), focusing on data from nodes with high SNR close to the injection site (Figure 6, Supplementary Figure S7). The interruption of fluid injection coincided with a flip of approximately 90$^\circ$ in $\varphi$ at the closest stations N23, N24, N25, N32 (Figure 6) and the appearance of the strike-slip event cluster (Figure 2). When injection resumed on July 1, we observed no distinct changes in seismicity or rotation in the dominant seismic anisotropy direction. Stations located further away from well KG-26 (e.g. N9) appear not to have been affected by the fluid injection interruption, as no changes were observed in the fast S-wave direction (Supplementary Figure S7).

**Figure 7:**
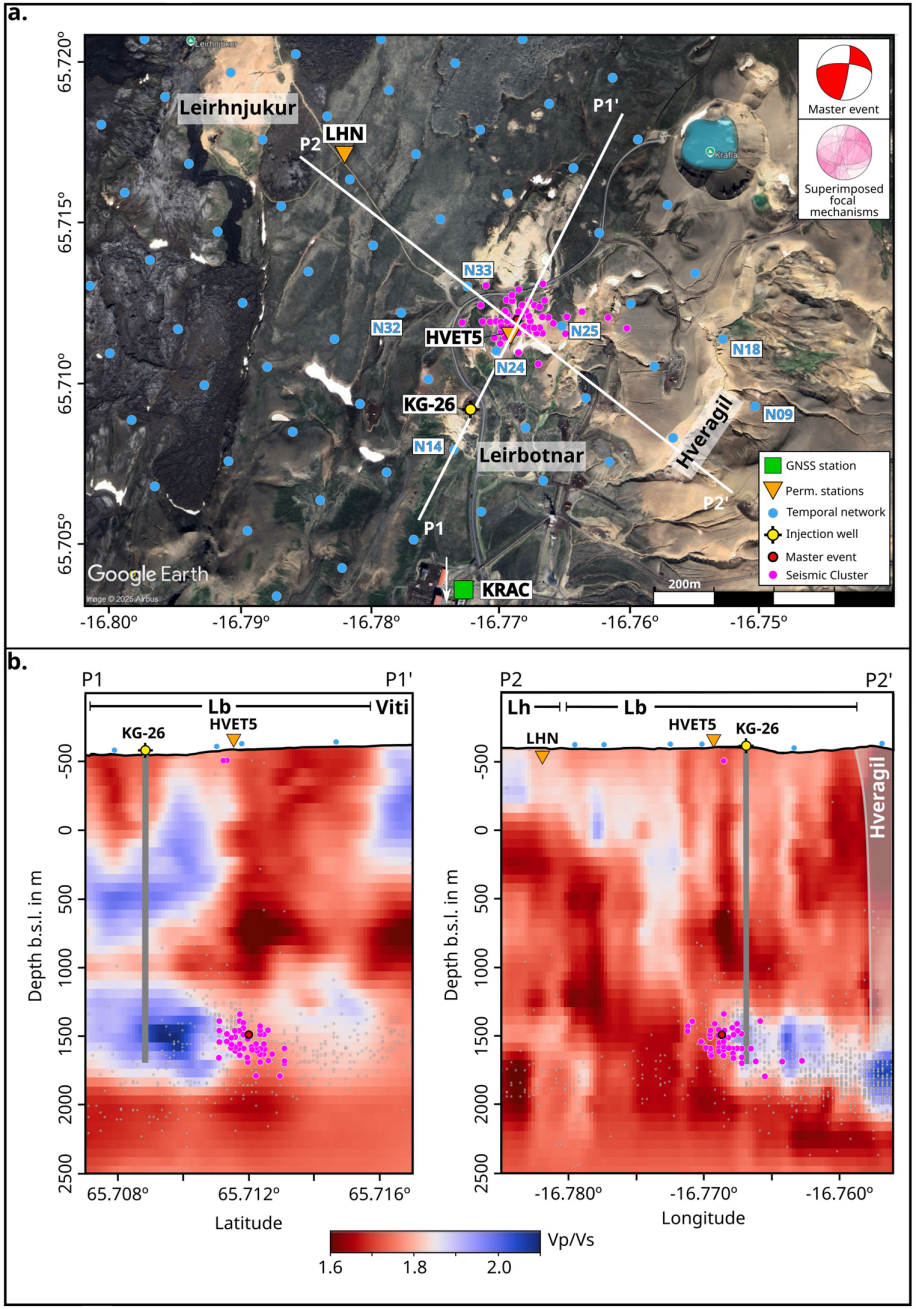
a) Satellite image (Google Earth^84^) with the location of the temporary network (light blue), the permanent stations (orange triangles), the injection well KG-26 (yellow circle), the arrow towards the GNSS station KRAC (green square - see location in Figure 1). The pink points are the strike-slip events found by template matching in the ÍSOR catalog, while the red point with black edge is the location of the master event. The focal mechanisms shown in the upper right corner are the master events and the superimposed focal mechanisms found by the template matching. P1 and P2 show the orientations of the cross-sections shown in (b). b) P1 and P2 cross-sections: $V_p/V_s$ ratios derived from local earthquake tomography^64^, grey points represent seismicity recorded between 2017 and 2022 within 200m of the cross-section and are shown to highlight the different spatial patterns of the overall seismicity compared to the strike-slip events. Vertical grey line, orientation of the well KG-26. Greyed out vertical structure in P2 shows the location of the Hveragil fissure. Abbreviations - Lh (Leirhnjúkur), Lb (Leirbotnar) geothermal subfields.

**Figure 8:**
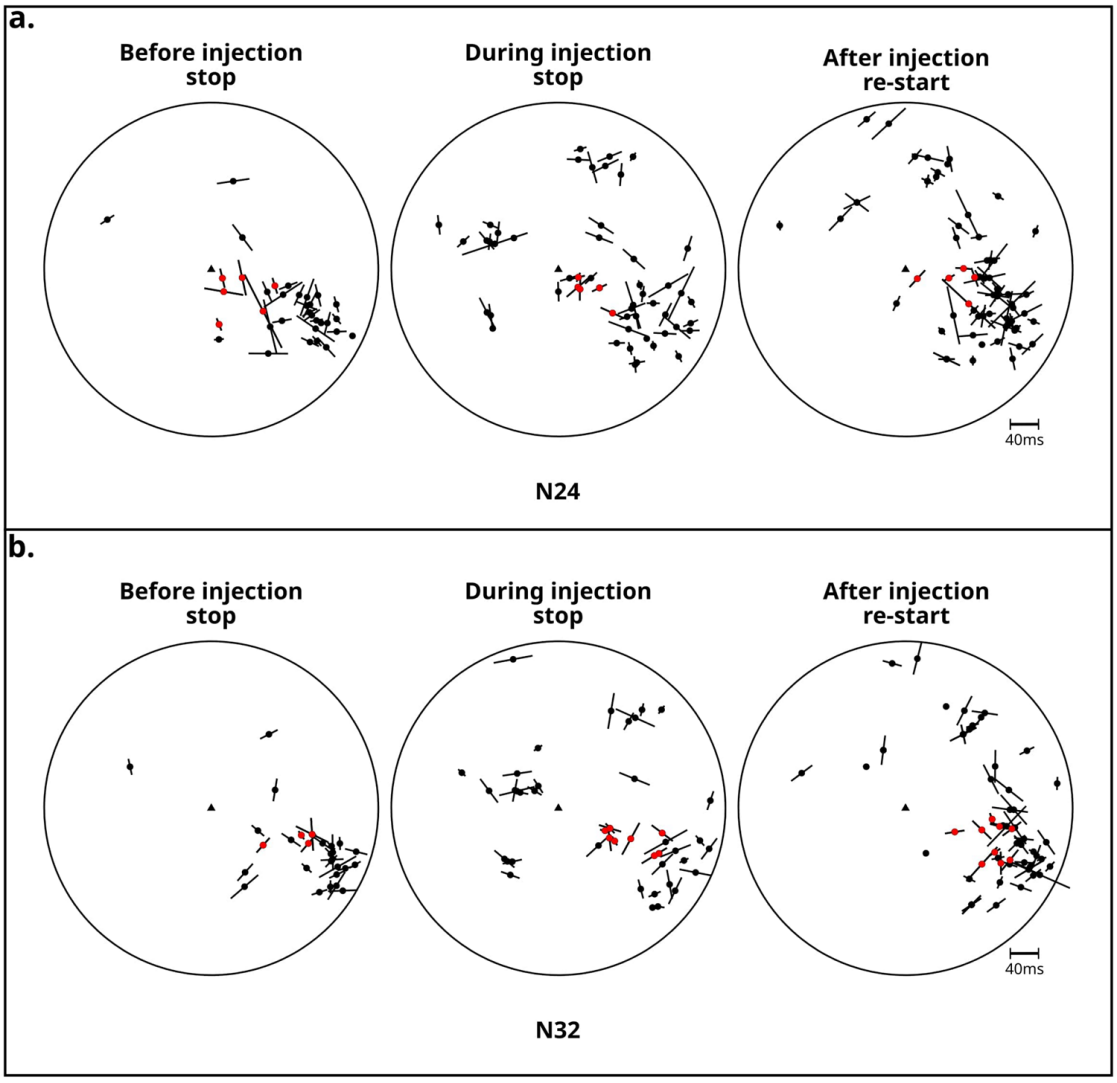
Polar equal-area projection for the fast S-wave polarization for the stations N24 (a) and N32 (b) showing $t_d$ (length of the bar through each event) and $\varphi$ (orientation of the bar) before, during and after the injection stop. The station is located in the center of each circle (black triangle) and the outline of the circle corresponds to the incidence angle of 45$^\circ$. Red events are those, which rays travel through the presumed water body (ray paths and the daily evolution of $t_d$ are shown in the Supplementary Figure S8, for the other polar equal-area projections see Supplementary Figure S9).

In order to delimit the area affected by the injected cold fluids, the Vp/Vs tomography results^64^ were used. At the bottom of KG-26 a high Vp/Vs volume of 500 m $\times$ 500 m $\times$ 500 m was identified. High Vp/Vs values and cold temperatures may reflect the presence of liquid water^64,73,74^). A closer examination of the rays which travel through the presumed water body (Figure 7) reveals, for most of them, flips up to 90$^\circ$ in the dominant direction of $\varphi$ and a reduction in the $t_d$ values, when the injection was interrupted (Figure 8, Supplementary Figure S8, Supplementary Figure S9). However it is important to note, that this is a conservative scenario, since the resolution of the tomography is 250 m and the actual volume affected by the injected water might be larger, with potentially more rays traveling through it.

We conclude that a high-resolution spatio-temporal SWS analysis allows the identification of changes in the seismic anisotropy during anthropogenic manipulations, with greater detail than the lower-density, large-scale network.

## Discussion

The application of different methods on multi-scale seismic datasets can track variations in seismicity, seismic anisotropy and stress field associated with the interruption and resumption of fluid injection. The occurrence of strike-slip events near the center of Krafla caldera (close to the injection well KG-26), in an extensional regime where normal faulting prevails^42^, highlights the complexity of the stress field at Krafla. Injection has been near-continuous since 2002 at well KG-26, which may be a cause for the nowadays observed stress conditions. Thus, this ”background loading” from fluids may be the reason why strike-slip events were only observed when injection was interrupted in 2017 and, above all, in 2022, with the more conservative 3 station TM analysis (stations LHN, SPB and HVET5 - Figure 2, top). Between 2018 and 2021, a correlation between injection shut-ins and strike-slip seismicity could be identified only with a less reliable single-station TM analysis at station HVET5, and not at network scale (Supplementary Figure S4). Those detections are potentially weaker strike-slip events and, thus, not included in the ÍSOR catalogue. The change in seismicity and waveform correlation patterns may be related to crustal rebound caused by reduced mass extraction^75^, observed in the GNSS stations in the central caldera (Figure 2, KRAC station - Up component). Therefore, changes may have occurred in the ”background loading” of the system. Overall, when relying solely on the strike-slip events included in the ÍSOR catalogue, the correlation between injection pauses and seismicity increases is, at best, weak. However, the combination of the long-term observations with the spatially dense temporary network deployed in 2022 allows us to identify previously undetected events, thereby revealing a robust correlation between anthropogenic activity and strike-slip events.

By applying template matching to the high-resolution dataset, we discovered that strike-slip events exhibit swarm-like behavior, with the frequency of earthquakes increasing almost instantaneously when injection is interrupted, similarly to what was observed with the single station TM at HVET5 (Supplementary Figure S4). This sharp increase in the number of events is followed by a gradual decrease, which is typical for a mainshock - aftershock sequence according to Omori’s law^76^ (Figure 2, bottom). The observed short-term correlation between seismicity and the stop of injection is potential evidence that these events are induced by anthropogenic activity, as previously observed at Krafla^17^ and elsewhere^8,12,13^. Given the strike-slip mechanism of the master event and the strike direction of one of its nodal planes, oriented WNW-ESE, we conclude that the interruption of fluid injection likely activates a single transform fault, which accommodates the shear strain associated with rifting at Krafla^54^. A similar reactivation of strike-slip faults has been observed in rifting areas during dike injection episodes^77–79^. The correlation between the interruption of fluid injection and faulting activity may indicate that human interventions change the stress field in the subsurface, thereby altering the seismicity patterns as well as seismic properties of the reservoir rock (i.e., the medium through which seismic waves propagate).

By comparing the event locations obtained through the long-term template matching analysis with the elevated Vp/Vs values^64^ and interpreted as the reservoir of the cold injected water, we observe that the hypocenters are located (with a vertical and horizontal uncertainty of 100 - 200 m) along the edge of this volume (Figure 7). Assuming that the pores in the high Vp/Vs area are saturated by fluids during injection, we anticipate a subsequent pore depressurization during the injection pause, which would lead to stress release and changes in the seismic anisotropy. This hypothesis is in agreement with the observed flips of $\varphi$ by 90$^\circ$ degrees and decreases of $t_d$ for the S-waves traveling through the area of the water body (Figure 8, Supplementary Figure S8, Supplementary Figure S9). The localized variation in seismic anisotropy, the backazimuth analyses of the incoming S-waves and the temporal correlation with changes in reinjection patterns, together with the absence of any reported volcanic activity during the analyzed period, are consistent with stress field rotation induced by the abrupt injection shut-off and likely rule out alternative causes of seismic anisotropy variation (e.g., in the lithologies themselves). Therefore, the anisotropy and $S_{Hmax}$ should instantaneously reflect the local tectonic conditions^31,43^. When injection resumed, we observed an increase in $t_d$. Although we expect a delayed change in $\varphi$ associated with slow pore pressurization, none was observed, probably due to the limited duration of our high-resolution seismic survey. This may indicate the delayed nature of pressurization due to the high permeability of the system^50,60^. However, at Krafla, changes in $t_d$ have been observed upon injection, but no changes in $\varphi$ were reported^18^ before the present study. Thus, we assume that longer high-resolution geophysical studies may be necessary to fully resolve the impact of fluid injection on stress and seismicity in the shallow subsurface.

**Figure 9:**
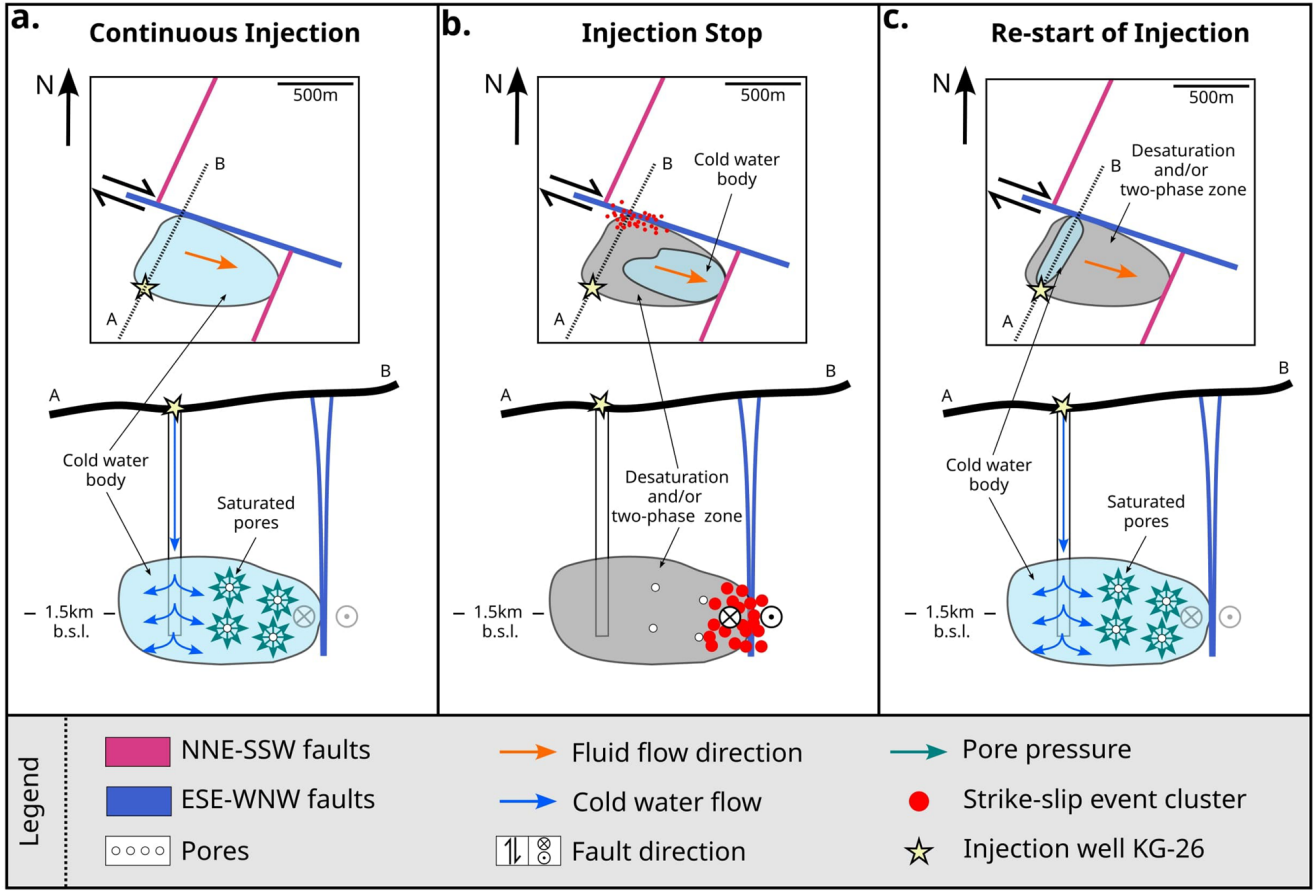
Conceptual model of the triggering mechanism behind the strike-slip events in the area of well KG-26. Primary normal faults (magenta), secondary transform fault (blue), direction of discharge (orange arrows), injection well (yellow star), blue arrows (fluid injection at the well), dark green arrows (isotropic orientation of the pore pressure), orientation of the fault movement (black arrows), red points (strike-slip events), blue area (cold water reservoir), grey area (desaturated area and/or two-phase conditions). a) Before the injection stop: Over-pressurization of pores stabilizes the system, b) During injection stop: Decreasing pore pressure allows the transform fault to generate brittle failure c) After re-starting the injection: Over-pressurization stabilizes the system, leading to seismic quiescence of the transform fault.

In conclusion, we observed that during periods of prolonged and constant injection of cold water in the Krafla reservoir, the system may establish a new ”background load” with a seemingly low occurrence of strike-slip events close to well KG-26. This could be due to the lubrication of the fault by the injected fluids^80,81^, which would allow a near-continuous, near-aseismic stress release (Figure 9a). When the injection is shut down, the observed flip of $\varphi$ and the decrease of $t_d$ indicate changes in the pore pressure, reducing the critical stress that must be exceeded to produce seismicity. Consequently, the pore pressure can no longer overprint the local stress field, resulting in the triggering of strike-slip events along one of the transform structures oriented along the WNW-ESE direction to accommodate the new dominant stress field (Figure 9b). The reduction in pore pressure may be due to drainage of the system or changes in the fluid phase, due to the high temperatures in the surrounding rocks (as recorded at the bottom of the IDDP-1 well^82^). However, a decrease in pore pressure would not lead to an increase - but rather a decrease in Coulomb stress, making the fault less likely to fail. The observed increase in seismicity and fault reactivation can instead be attributed to changes in differential stress induced by the abrupt injection shutdown, leading to elevated Coulomb stress on the fault^19,20,83^. Therefore, we infer that the reduction of pressure due to the injection stop is the primary driver of fault reactivation, with the associated increase in normal stress outweighing the effect of the pore pressure decrease. When injection resumed, we anticipate that the reservoir would progressively become pressurized, likely re-establishing the original ”background load” and overprinting the tectonic stress (Figure 9c).

This study underlines the importance of adopting a multi-parametric approach to better understand the impacts of geothermal operation on stress field and induced micro-seismicity in and around a geothermal reservoir. Krafla is one of the few sites where we can study how fluid-saturated micro-cracks and micro-pores evolve under changing stress conditions, including the rarely observed 90$^\circ$ flip of the fast S-wave polarization. In addition, it highlights the susceptibility of the regional stress field to anthropogenic geothermal activity, providing a fundamental understanding necessary for the safe use of subsurface resources in volcanic areas.

## Data and methods

### Seismicity

For the long-term analysis, a pre-existing event catalog^42,64^ was used. This catalog was used as a database for the analysis of seismicity patterns and a selection of it for the long-term SWS analysis. For the short-term analysis, only the data of the temporarily installed 98 3-component nodes were used. After removing false detections, 404 events identified by the small-scale network were located by using waveform coherency. Only data from the temporary nodal deployment were considered for this, since all the nodes have the same instrument response and the waveforms are comparable. The LOKI code^85^ was used for the location analysis and was applied to all events detected by this network, including events that are already listed in the catalog^42^. As input, the 7 second waveform snippets of the 3 components, filtered between 1 and 40 Hz, and high-resolution 3D P- and S-wave velocity models^64^ are used. The grid point where LOKI detects the highest coherency value is considered as the origin of the event. Locations of events calculated with LOKI are on average 500 m deeper than the ones in the ÍSOR catalog. This is probably due to the fact that no data from the remote, permanent stations was used in this localization process, to avoid combining data from different sensor types for the waveform coherency analysis. However, locations obtained with LOKI can be used for the SWS analysis, since the differences are still within the error margin of the earthquake location. An example for the localization of one event and the location for all the events of the dense catalog is given in Supplementary Figure S2.

### Template matching

Template matching was carried out using obspy with the same event as reference trace (ÍSOR ID 2022mprrpt - June 28, 12:16:50) on the long- and short-term datasets. For both, the raw data were filtered between 1 and 20 Hz. For the long-term analysis, the template matching was carried out on three permanent stations (LHN, SPB and HVET5), where a detection was labeled as of a similar source as the master event if the combined cc was above 1.95 (theoretical maximum of the summed cc: 3) and if the event was listed in the ÍSOR catalog. For the short-term analysis, template matching was only carried out on the node N24, because this station presents a higher SNR than the others in the area and it was located directly above the assumed strike-slip cluster. Here, the detection of a strike-slip event was considered true, if the cc was higher than 0.5, even if the event was not previously listed in any catalog.

### Shear-wave splitting and ray tracing

In order to define the seismic anisotropy of the upper crust in the Krafla caldera, the shear-wave splitting phenomenon was examined with an open-source software designed for local shear-wave splitting studies, called Pytheas^86^. The velocity model used for this study is the layered 1D model provided by ÍSOR^87^. The events analyzed for both the permanent network and the nodal array, were filtered by incidence angle ($45^{\circ }$), and a band-pass filter was applied either between 1 Hz and 20 Hz or between 3 Hz and 30 Hz, depending on the frequency content of the event. For this study, only events with a clear impulsive S-wave arrival were considered to ensure that what we observe and measure is the first S-wave arrival. In addition, a polarigram and a hodogram displayed by the software were used to facilitate the picking process. The permanent network stations operated at a sampling rate of 200 Hz, resulting in time-delay measurements with a resolution of 5 ms, whereas the temporary stations, with a sampling rate of 250 Hz, allowed measurements in 4 ms increments. Since the picking was performed manually, the uncertainties of both, $\varphi$ and $t_d$ can not be quantitatively assessed due to human bias^86^. However, a quality grade ranging from A to D was assigned to each pick, with A being the best and D being the ones which had more than one possible outcome or lacking a perfect fit^88^. The distribution of grades assigned to picks from stations of both the permanent and the temporary networks is provided in Supplementary Figure S10. Supplementary Figures S11 - S15 present examples of picks in the Pytheas software for each grade. In Krafla, the registered waves are not always purely tectonic, with noise levels being high, and the waveforms not being always clear. For that reason, all events with grades from A to D were used for the purpose of the present study. Two more grades exist for lower quality events (E and N), but those were not considered in our interpretation.

For the small-scale, high-resolution SWS analysis, we used events that were both detected and located with the temporal nodal array (Figure 5a), applying the same constraints to the source-receiver geometry and SNR as in the large-scale SWS analysis. At first, a random selection of events was picked from all the nodes. After identifying the eight most favorable nodes with the best SNR and shortest temporal variability (N23, N24, N25, N32, N33, N16, N18 and N9), we increased the data picking density (Figure 5a, c)

The ray tracing to identify the station-event pairs which rays sampled the water body emplaced by the continuous injection at KG-26, was carried out using the finite difference eikonal solver by Podvin and Lecomte (1991)^89^ that is implemented in the local earthquake tomography code TomoTV^90,91^. For this step the subsurface is discretized in grid cells of 50 m and the ray is traced from the source to the station with the high resolution 3D velocity model^64^. The water body around KG-26 was identified as the volume where the Vp/Vs ratio is higher than 1.85. Thus, the estimated extent of the water body is 500 m $\times$ 500 m $\times$ 500 m.

## Supplementary Information

Supplementary Information.

## Data Availability

The continuous raw data of the large-scale network are available from Landsvirkjun but restrictions apply to the availability of these data, which were used under license for the current study, and so are not publicly available. Data are however available from the corresponding authors upon reasonable request and with permission of Landsvirkjun. A subset of the data snippets (7 second in .mseed format for the events in 2021 - 2022, large- and small scale network), that have been used for the picking of the fast and slow S-wave onset are included in Glück at al. (2025). The results of the manual picks (φ and td) and the continuous data of the Z-component of node N24 are available in the Zenodo repository (doi:10.5281/zenodo.16894876).
