# Supplementary material for: Fluid injection interruption causes temporary changes in local stress field and induced seismicity at Krafla caldera, Iceland
Source: Sci Rep. 2026 Feb 26;16:7942. doi: 10.1038/s41598-026-39532-1 (PMC12953912; doi:10.1038/s41598-026-39532-1)
Supplement: Supplementary file 1 — Supplementary Information. [file 41598_2026_39532_MOESM1_ESM.pdf]

Supplementary Materials for

Fluid injection interruption causes temporary changes in local stress field and induced seismicity at Krafla caldera, Iceland

Elisabeth Glück[1†], Roberto Davoli[2*†], Thorbjörg Ágústsdóttir[3],Stéphane Garambois[1], Egill Árni Gudnason[3], Yan Lavallée[2], Anette K. Mortensen[4], Bettina Scheu[2], Jean Vandemeulebrouck[1]

[*]Corresponding author. Email: roberto.davoli@lmu.de

[†]These authors contributed equally to this work.

This PDF file includes:

Figures S1 to S15

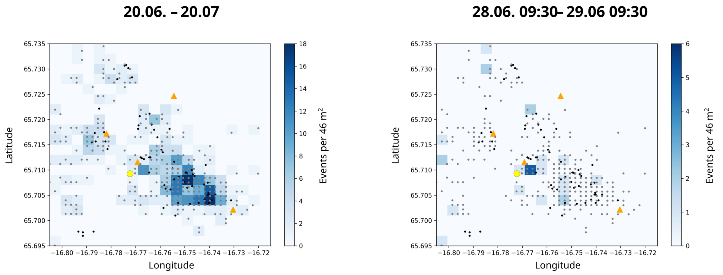

Figure S1: **2D histogram of the localisation of the additional detections with the nodal array.** Left the whole time span, right the first 24h after the reinjection stop (yellow circle-well K26). A clear anomalous increase in the number of events can be observed in the right figure close to K26, where the 24h after the stop are shown.

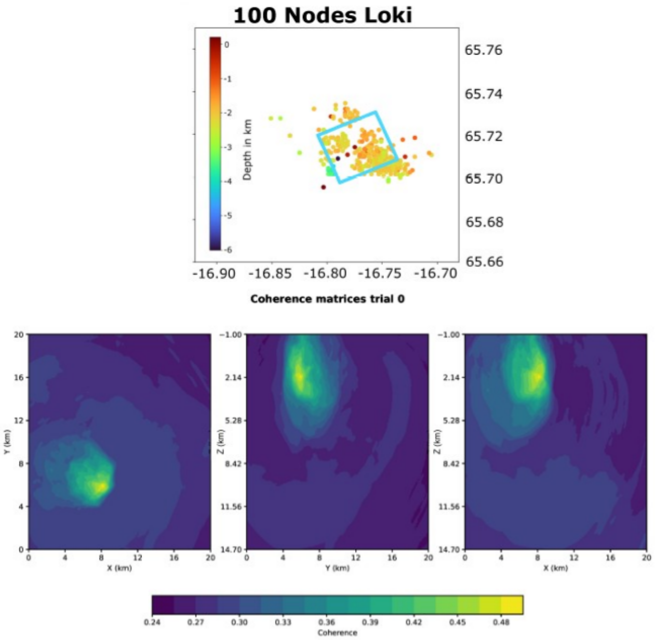

Figure S2: **LOKI Localisations from waveform coherency analysis.** LOKI Localisations, blue triangle is the extent of the nodal array and an example of cross sections (local coordinate system: origin 411376 mE/7282985 mN, UTM zone 28N) through the coherency matrix of one event (2022-06-28 02:27:11.86, ISOR event ID: isor2022moydnh)

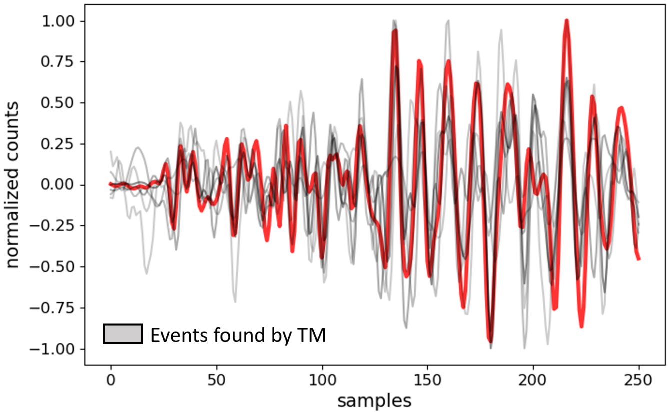

Figure S3: **Normalized waveform of the Master Event at node N24 (red) and normalized waveforms of the events found by TM (grey).**

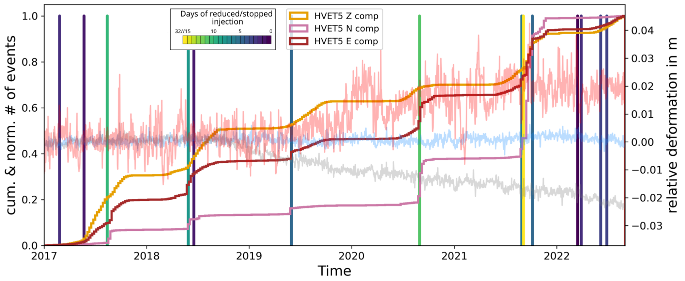

Figure S4: **Template-matching results for the master event from permanent station HVET5.** The long-term evolution of seismicity patterns of the strike-slip events was obtained by template matching using a relatively low correlation coefficient threshold of 0.5, applied independently to all 3 components, which may contain a substantial number of false detections. The figure also includes the three components of the GNSS station KRAC (light red line indicates the Up component, light blue line indicates the East component and light grey line indicates the North component; from Yang et al., 2023) and the timing of injection pauses at well KG-26 as in Figure 2.

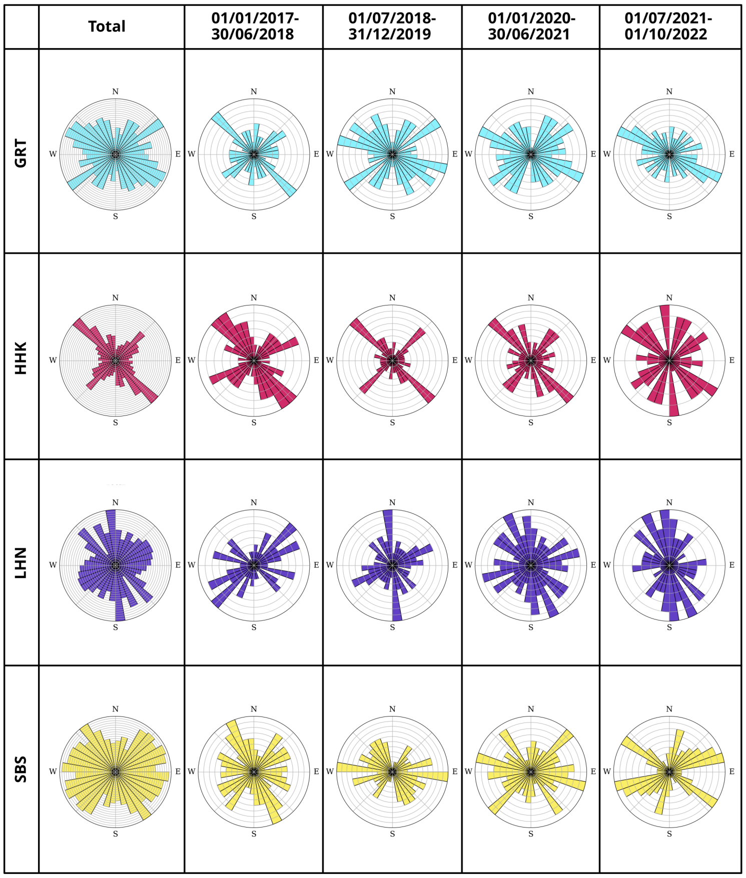

Figure S5: **Timelapse of the SWS result for the remaining stations of the permanent network.** Time-lapse in 1.5 year steps of the general stress field at the permanent stations HVET5 and SPB. The binning in the diagram is 10° and each circular grid line equals 1 pick.

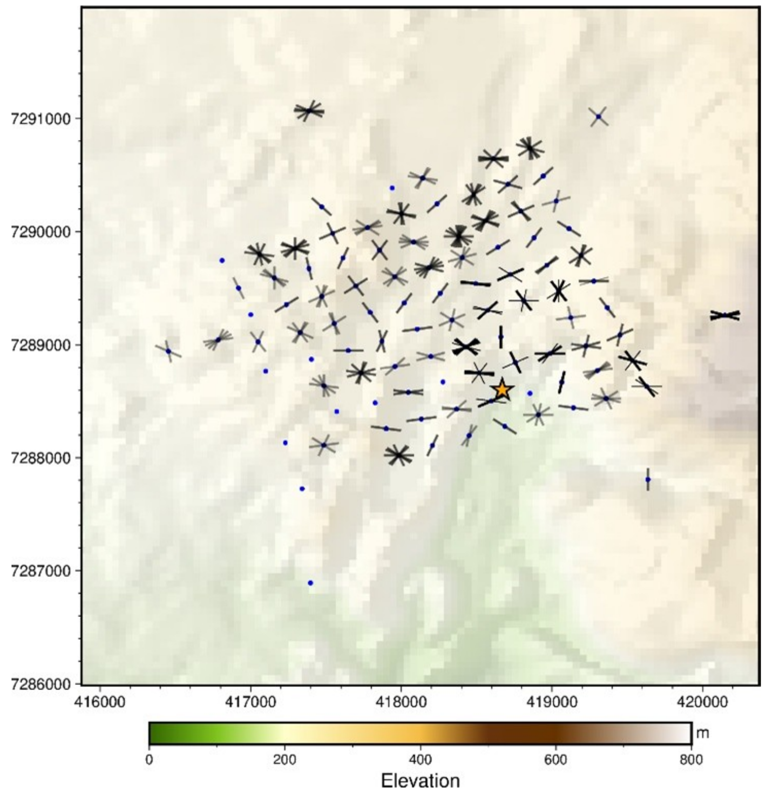

Figure S6: **Main orientation of φ for all the nodes where the shear waves were picked.** Orange star: Injection well KG-26, blue points: seismic nodes, black lines: orientation of φ. The map was generated with PyGMT (Tian et al., 2026).

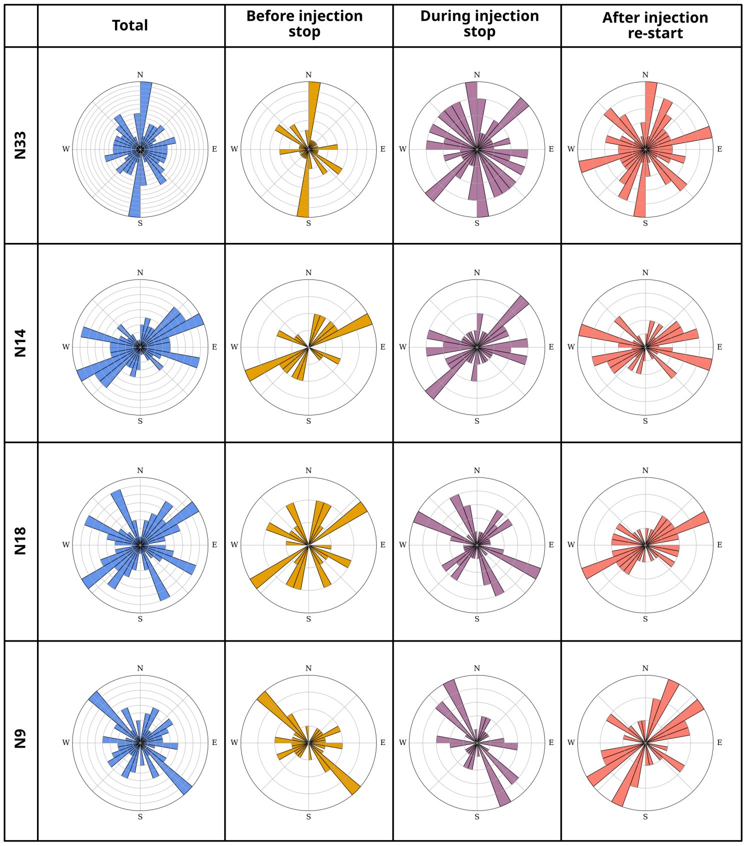

Figure S7: **Timelapse of the SWS result of the small-scale experiment for the remaining stations around well KG-26 and for some that are located further away.** The binning in the diagram is 10° and each circular grid line equals 1 pick.

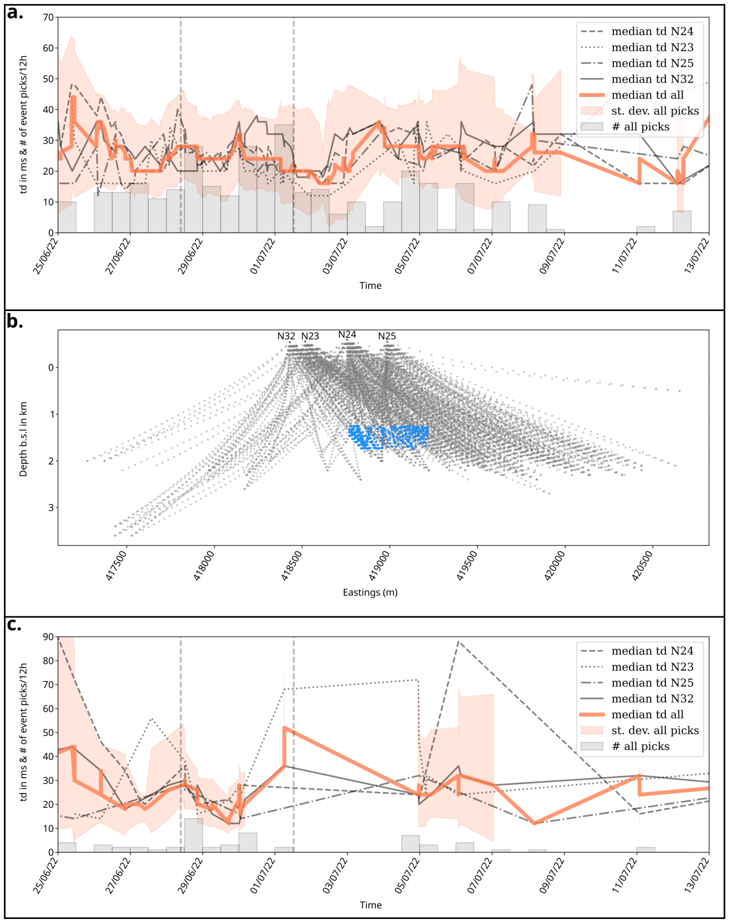

Figure S8: **Evolution of t_d and ray tracing. (a)** t_d evolution during the course of the high resolution experiment for all the picked events in stations N23, N24, N25 and N32; **(b)** ray paths (grey) of events picked at stations N23, N24, N25 and N32. In blue the area where the rays sampled the supposed water body; **(c)** t_d evolution of the high resolution experiment for the events that sample the water body picked at stations N23, N24, N25 and N32.

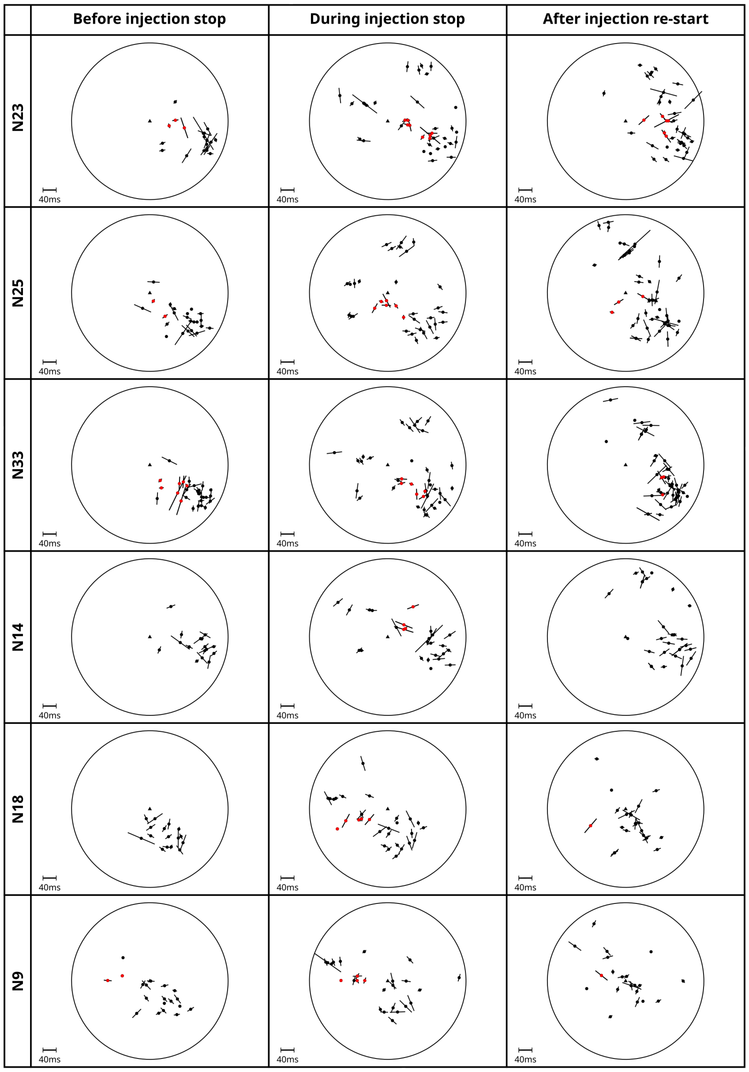

Figure S9: **Polar equal-area projection for the fast S-wave polarization for the stations N23, N25, N33, N14, N18 and N9.** Showing t_d (length of the bar through each event) and phi (orientation of the bar) before, during and after the injection stop. The station is located in the center of each circle (black triangle) and the outline of the circle corresponds to the incidence angle of 45°. Red events are those, which rays travel through the presumed water body.

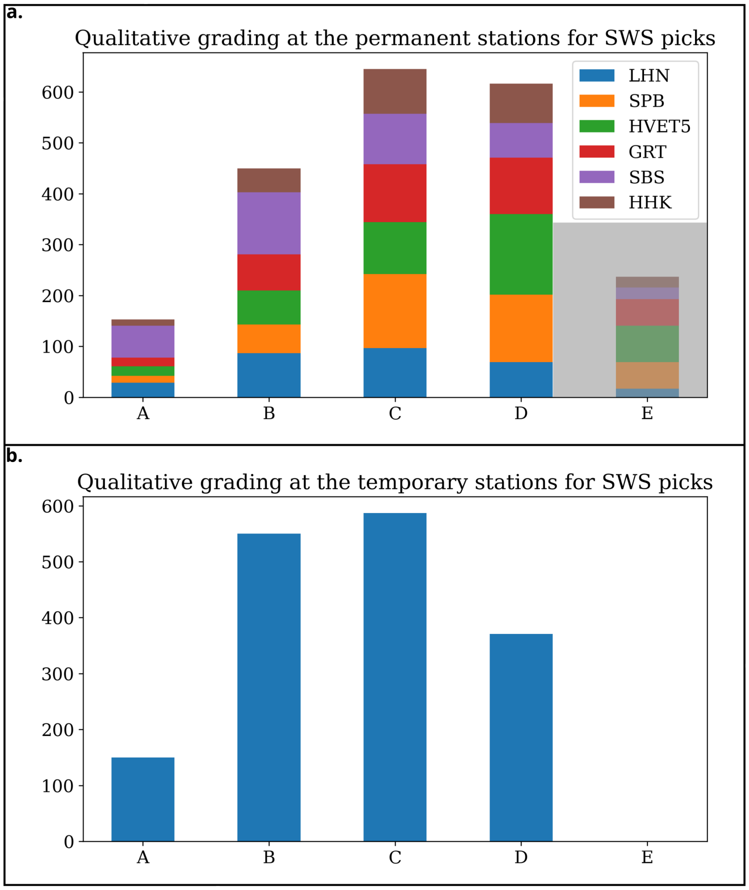

Figure S10: **Qualitative assesment of the SWS picks.** Distribution of the grades associated with the manual SWS picks in Pytheas,: a) permanent network with a subdivision by station, b) temporary network. Grade A provides the best picks, and the quality decreases alphabetically (with D being the lowest quality used picks); Picks with grade E (grey area) were not included in the analyses. A detailed guideline for performing the qualitative grading is provided in Kaviris et al. (2018).

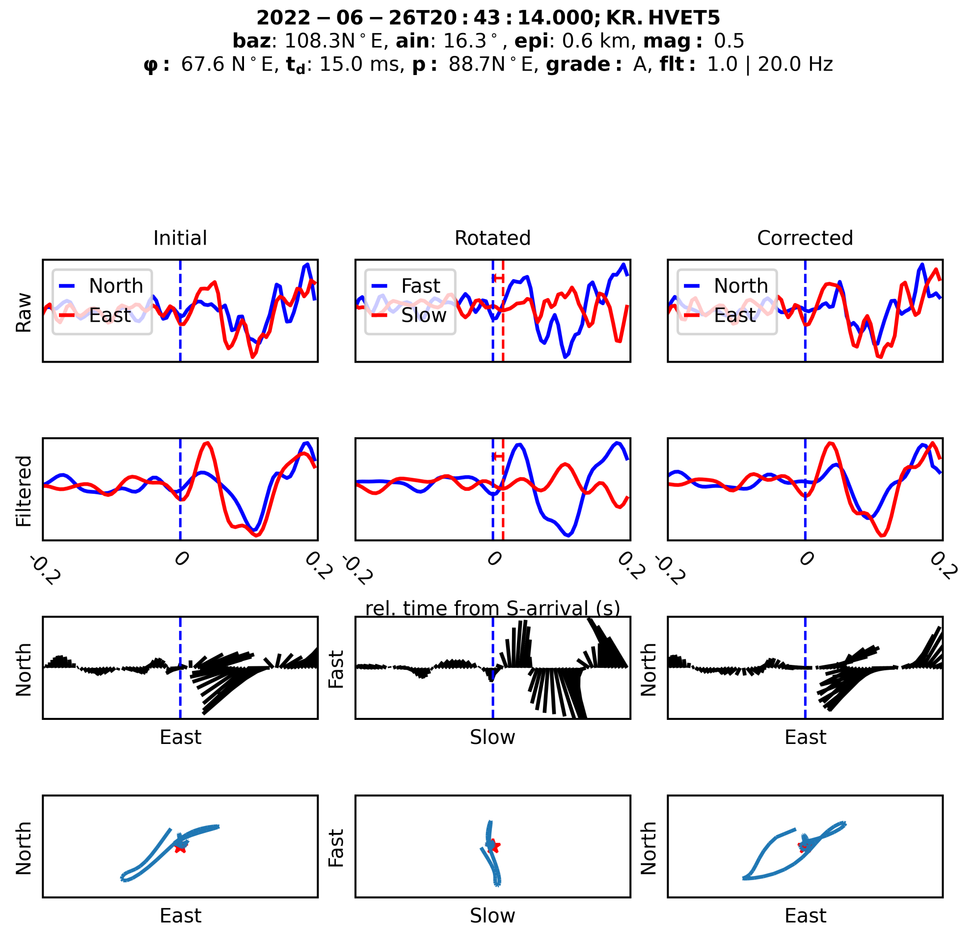

Figure S11: **Example of the stages of a measurement with the Pytheas software for picks with Grade A.** Columns: 1 - Initial NE-system of the waveform, 2 - Rotated waveforms in Fast-Slow system with the correction for anisotropy, 3 - Corrected waveforms in NE-system; Rows: 1 - Raw waveforms, 2 - Filtered waveform (1-20 Hz), 3 - Polarigram for each step, 4 - Hodogram showing the particle motion for each step. The blue dashed line indicates the manually picked first arrival pick of the S-wave, whereas the red dashed line in the “Rotated” stage is automatically determined by the software based on the time delay between the fast and slow S-wave components. It is essential for the analyst to iteratively review the different stages to ensure the validity of the results and to select the optimal fit.

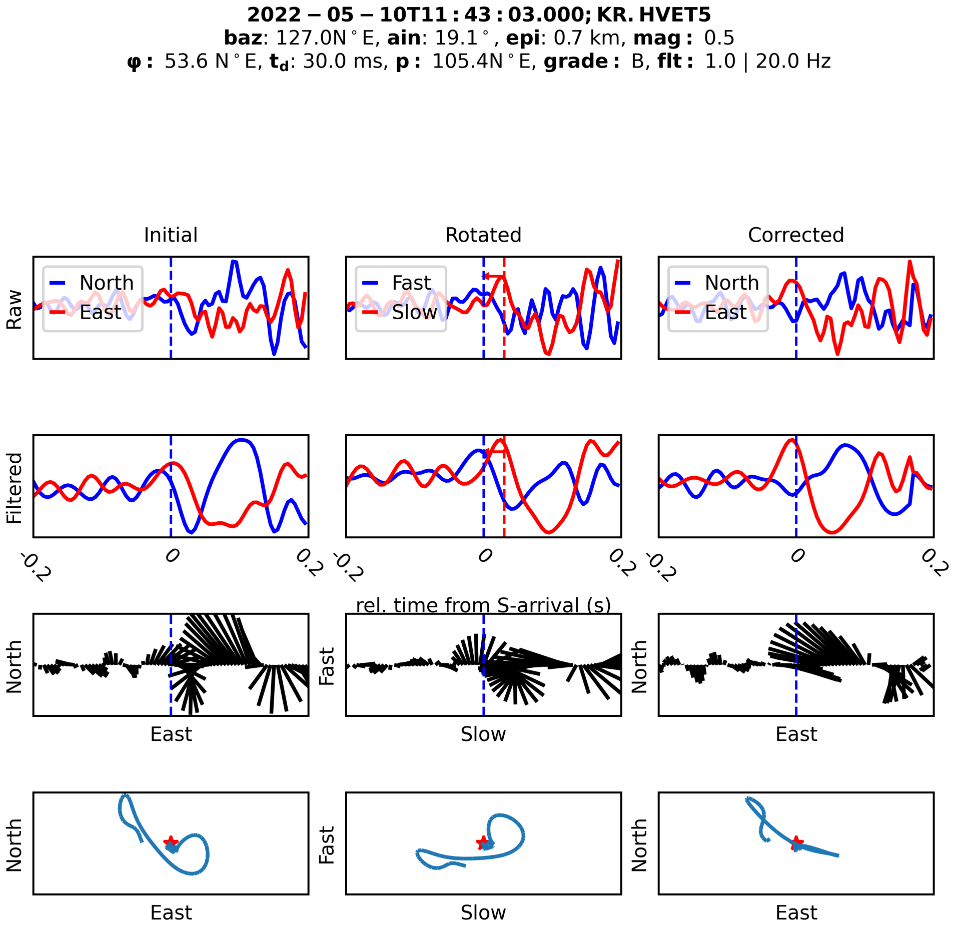

Figure S12: **Example of the stages of a measurement with the Pytheas software for picks with Grade B.** Columns: 1 - Initial NE-system of the waveform, 2 - Rotated waveforms in Fast-Slow system with the correction for anisotropy, 3 - Corrected waveforms in NE-system; Rows: 1 - Raw waveforms, 2 - Filtered waveform (1-20 Hz), 3 - Polarigram for each step, 4 - Hodogram showing the particle motion for each step. The blue dashed line indicates the manually picked first arrival pick of the S-wave, whereas the red dashed line in the "Rotated" stage is automatically determined by the software based on the time delay between the fast and slow S-wave components. It is essential for the analyst to iteratively review the different stages to ensure the validity of the results and to select the optimal fit.

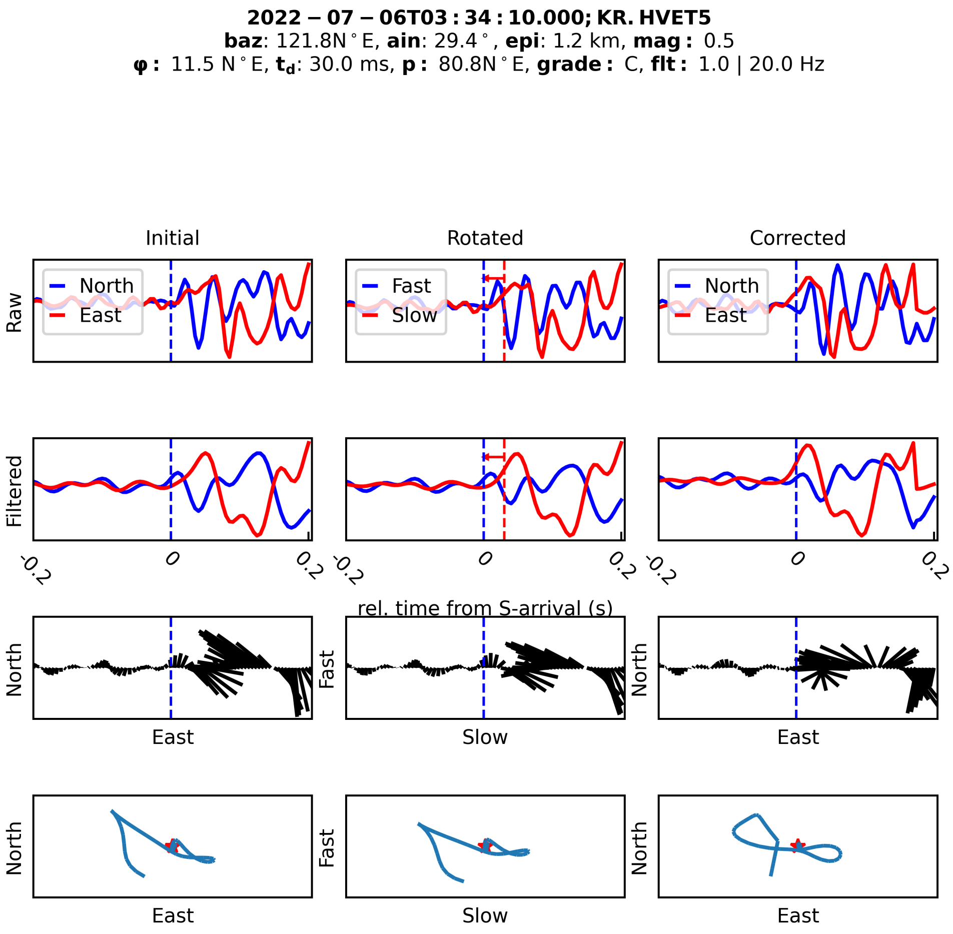

Figure S13: **Example of the stages of a measurement with the Pytheas software for picks with Grade C.** Columns: 1 - Initial NE-system of the waveform, 2 - Rotated waveforms in Fast-Slow system with the correction for anisotropy, 3 - Corrected waveforms in NE-system; Rows: 1 - Raw waveforms, 2 - Filtered waveform (1-20 Hz), 3 - Polarigram for each step, 4 - Hodogram showing the particle motion for each step. The blue dashed line indicates the manually picked first arrival pick of the S-wave, whereas the red dashed line in the "Rotated" stage is automatically determined by the software based on the time delay between the fast and slow S-wave components. It is essential for the analyst to iteratively review the different stages to ensure the validity of the results and to select the optimal fit.

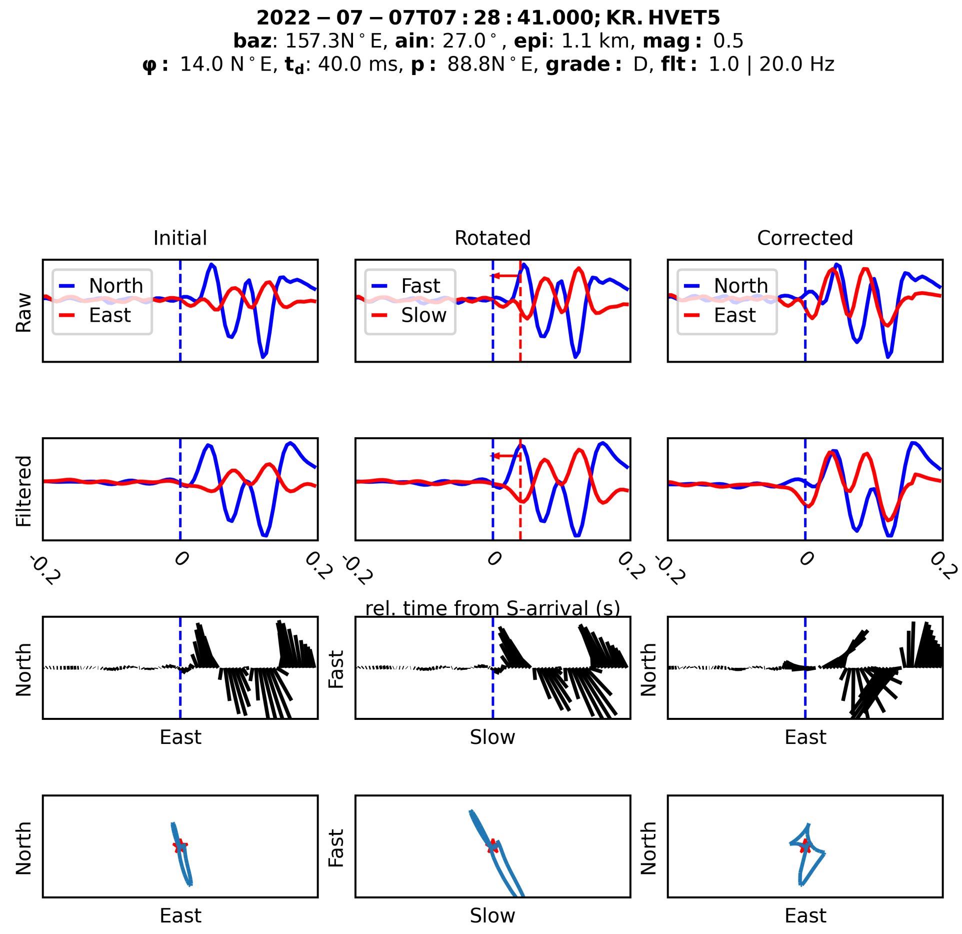

Figure S14: **Example of the stages of a measurement with the Pytheas software for picks with Grade D.** Columns: 1 - Initial NE-system of the waveform, 2 - Rotated waveforms in Fast-Slow system with the correction for anisotropy, 3 - Corrected waveforms in NE-system; Rows: 1 - Raw waveforms, 2 - Filtered waveform (1-20 Hz), 3 - Polarigram for each step, 4 - Hodogram showing the particle motion for each step. The blue dashed line indicates the manually picked first arrival pick of the S-wave, whereas the red dashed line in the "Rotated" stage is automatically determined by the software based on the time delay between the fast and slow S-wave components. It is essential for the analyst to iteratively review the different stages to ensure the validity of the results and to select the optimal fit.

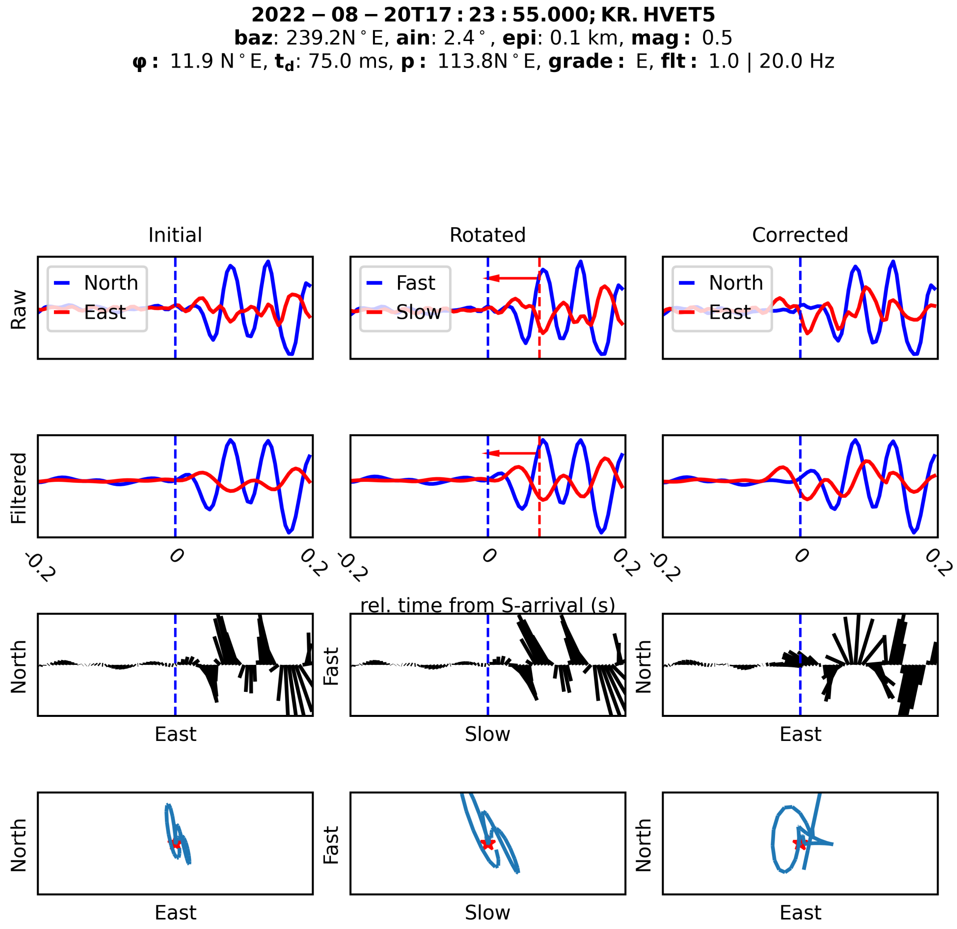

Figure S15: **Example of the stages of a measurement with the Pytheas software for picks with Grade E.** Columns: 1 - Initial NE-system of the waveform, 2 - Rotated waveforms in Fast-Slow system with the correction for anisotropy, 3 - Corrected waveforms in NE-system; Rows: 1 - Raw waveforms, 2 - Filtered waveform (1-20 Hz), 3 - Polarigram for each step, 4 - Hodogram showing the particle motion for each step. The blue dashed line indicates the manually picked first arrival pick of the S-wave, whereas the red dashed line in the "Rotated" stage is automatically determined by the software based on the time delay between the fast and slow S-wave components. It is essential for the analyst to iteratively review the different stages to ensure the validity of the results and to select the optimal fit.
